# A Computational, Tissue-Realistic Model of Pressure Ulcer Formation in Individuals with Spinal Cord Injury

**DOI:** 10.1371/journal.pcbi.1004309

**Published:** 2015-06-25

**Authors:** Cordelia Ziraldo, Alexey Solovyev, Ana Allegretti, Shilpa Krishnan, M. Kristi Henzel, Gwendolyn A. Sowa, David Brienza, Gary An, Qi Mi, Yoram Vodovotz

**Affiliations:** 1 Department of Computational and Systems Biology, University of Pittsburgh, Pittsburgh, Pennsylvania, United States of America; 2 Joint PhD Program in Computational Biology, University of Pittsburgh and Carnegie Mellon University, Pittsburgh, Pennsylvania, United States of America; 3 Center for Inflammation and Regenerative Modeling, McGowan Institute for Regenerative Medicine, University of Pittsburgh, Pittsburgh, Pennsylvania, United States of America; 4 Department of Mathematics, University of Pittsburgh, Pittsburgh, Pennsylvania, United States of America; 5 Department of Rehabilitation Science and Technology, School of Health and Rehabilitation Science, University of Pittsburgh, Pittsburgh, Pennsylvania, United States of America; 6 Spinal Cord Injury/Disorders Center, Louis Stokes Cleveland Veterans Affairs Medical Center, Cleveland, Ohio, United States of America; 7 Department of Physical Medicine and Rehabilitation, University of Pittsburgh School of Medicine, Pittsburgh, Pennsylvania, United States of America; 8 McGowan Institute for Regenerative Medicine, University of Pittsburgh, Pittsburgh, Pennsylvania, United States of America; 9 Department of Bioengineering, University of Pittsburgh, Pittsburgh, Pennsylvania, United States of America; 10 Department of Surgery, University of Chicago, Chicago, Illinois, United States of America; 11 Department of Sports Medicine and Nutrition, University of Pittsburgh, Pittsburgh, Pennsylvania, United States of America; 12 Department of Surgery, University of Pittsburgh, Pittsburgh, Pennsylvania, United States of America; Johns Hopkins University, UNITED STATES

## Abstract

People with spinal cord injury (SCI) are predisposed to pressure ulcers (PU). PU remain a significant burden in cost of care and quality of life despite improved mechanistic understanding and advanced interventions. An agent-based model (ABM) of ischemia/reperfusion-induced inflammation and PU (the PUABM) was created, calibrated to serial images of post-SCI PU, and used to investigate potential treatments *in silico*. Tissue-level features of the PUABM recapitulated visual patterns of ulcer formation in individuals with SCI. These morphological features, along with simulated cell counts and mediator concentrations, suggested that the influence of inflammatory dynamics caused simulations to be committed to “better” vs. “worse” outcomes by 4 days of simulated time and prior to ulcer formation. Sensitivity analysis of model parameters suggested that increasing oxygen availability would reduce PU incidence. Using the PUABM, *in silico* trials of anti-inflammatory treatments such as corticosteroids and a neutralizing antibody targeted at Damage-Associated Molecular Pattern molecules (DAMPs) suggested that, at best, early application at a sufficiently high dose could attenuate local inflammation and reduce pressure-associated tissue damage, but could not reduce PU incidence. The PUABM thus shows promise as an adjunct for mechanistic understanding, diagnosis, and design of therapies in the setting of PU.

## Author Summary

A virtual pressure ulcer was created as a platform to test therapies and determine the mechanisms most correlated with unfavorable outcomes. A layer of tissue fed with oxygen and diffusible molecules via blood vessels could develop an ulcer if pressure was applied, by simulating constriction of blood vessels in a circular region. Simulated ulcers were visually similar to digital photographs of ulcers in individuals with spinal cord injury in their irregular shapes, jagged edges, and overall progression in time. Statistical analyses of simulation outputs revealed that inflammation was an important determinant of ulcer severity and overall tissue damage. However, simulated clinical trials revealed that blocking the negative effects of inflammation could not prevent ulceration, and in order to be beneficial at all for this specific type of ulcer, anti-inflammatory treatments must be applied during the earliest stages of ulcer formation—before many clinical signs of ulceration appear.

## Introduction

Pressure ulcers (PU) affect 2.5 million US acute care patients and cost up to $1 billion per year [1]. They are a significant source of morbidity in both hospitalized patients and community-dwelling individuals with impaired mobility. PUs are especially common in individuals with spinal cord injury (SCI), occurring in up to 80% of this population at some point during their lifetime [2]. Spinal cord injury is a condition associated with decreased functional mobility, acutely increased oxidative activity in leukocytes, and chronic elevation of systemic inflammatory markers [3–5]. Pressure ulcers are thought to arise from pressure-induced ischemia, reperfusion injury, and/or deformation-induced cellular damage [6].

The pathogenesis of PU involves activation of the acute inflammatory response [7,8], a highly conserved cascade of events mediated by a set of specialized cells (e.g. platelets, mast cells, macrophages, and neutrophils) and molecules (inflammatory cytokines, free radicals, and Damage-Associated Molecular Pattern molecules [DAMPs]) that demarcate stressed or damaged tissue, and alert and recruit other cells and molecules. The inflammatory response can either restore the tissue to equilibrium (healing) and resolve, or become self-maintaining inflammation that causes and is caused by ancillary tissue damage. This excessive inflammation prevents the body from initiating wound healing and can lead to PU incidence [9]. The dynamic interplay of the inflammatory and healing cascades determines the ultimate success or failure of the healing process. These intracellular signaling networks and their products, including diffusible molecular mediators, are possible targets for diagnosis or therapeutic intervention. However, the complexity of the process as a whole, and the dependence of any given pathway or mediator on timing and context, complicates such translational approaches.

The “translational dilemma” centers on the inability of traditional, reductionist approaches to yield better diagnostics and novel drug targets for complex diseases [10–12]. Despite increased understanding of the underlying mechanisms and improved clinical vigilance, PUs remain a prevalent problem in hospitalized patients and people with chronic conditions such as diabetes and SCI [13–15]. While wound healing is well studied in animal systems [16–18], these animal models generally do not recapitulate the complex etiology of impaired wound healing such as that which occurs in PU. Only recently have experimental methodologies emerged that may allow for the study of the time courses of wound healing in humans [17], but these approaches are limited in that time courses of primary samples from humans with chronic wounds are difficult to collect without disturbing the very process being measured. As an alternative diagnostic approach, digital photographs of developing ulcers are, in theory, both plentiful and non-invasive—unlike wound biopsies.

Given the dire need for new therapeutic avenues for complex diseases, a platform combining *in silico* approaches with easily- and inexpensively-obtained clinical samples (such as photographic images), may yield novel diagnostic and therapeutic modalities. We hypothesize that wound images could be used to calibrate mechanistic simulations of inflammation and healing that could then be interrogated to predict how and when a small irritation might resolve or progress to become a chronic ulcer. Agent-Based Models (ABMs) allow for the investigation of both space- and time-dependent dynamics of complex systems via mechanistic simulations. The modeler provides behavioral rules that allow the model to proceed stepwise through discrete space and time. Unlike differential equation models, ABM simulations are stochastic and thereby able to replicate the randomness of biological processes. Furthermore, ABMs produce visual outputs whose morphological features evolve throughout a simulation and provide a rich set of spatio-temporal data that can be leveraged to probe underlying dynamics [19].

We report herein on the creation of an ABM of post-SCI PU formation (the Pressure Ulcer Agent Based Model, or PUABM) that incorporates key inflammation mechanisms. Explicitly included is the forward feedback loop of inflammation to damage to inflammation that has served as the core motif of our prior simulations of inflammation in both systemic and local contexts [9,20,21]. The PUABM replicates visual morphology associated with the development and resolution of post-SCI PU by simulating vascularized soft tissue overlaying a bony prominence (the clinically recognized “pressure points” at which PU typically develop) and the effects of repeated ischemia/reperfusion (representing the turning of a person with SCI in bed) on such an area of tissue. Also recapitulating clinical outcomes, the model reaches two distinct endpoints when simulated from the same initial parameters. We leverage thousands of model simulations to explore the root of this phenomenon and predict at what time an ulcer's fate is determined. We also demonstrate the utility of the PUABM as a platform for *in silico* clinical trials of strategies for prevention and therapies for treatment of PU post-SCI. This model suggests that the reason treatments thus far have been ineffective is that they have been applied too late.

## Materials and Methods

### Ethics statement

This study was approved by the University of Pittsburgh Institutional Review Board, approval number PRO08010011. Written informed consent was obtained from subjects participating in the study. If the subject was unable to provide written consent due to medical condition, proxy consent was obtained from the subject’s authorized representative, and the subject provided written consent for continued research participation as soon as they were able to do so.

### Clinical data collection

Photographic data were obtained from pressure ulcers sustained by patients enrolled in the Rehabilitation Engineering Research Center on Spinal Cord Injury at the University of Pittsburgh. Subjects were recruited from a single tertiary care center if they were 18 years of age or older and had sustained a traumatic spinal cord injury. Subjects were excluded if they had a history of pre-existing diseases that affect the inflammatory response to SCI (e.g. autoimmune disease, demyelinating diseases) or a history of previous SCI or other neurological diseases affecting motor and sensory function. Pressure ulcers were initially photographed when identified, and serial photographs were taken three times per week while patients remained in the acute care and/or weekly while in the inpatient rehabilitation hospital to monitor progression and/or healing, and during their outpatient visits or 6 month intervals after discharge. Photos were obtained using a Canon Power Shot SD 750 camera with 3X optical zoom lens with flash enabled, at a distance of 12 inches from the ulcer, resolution of 2048 x 1536 pixels. Pressure ulcer site, severity (stage 1–4, unstageable, or deep tissue injury), general appearance, size and shape were also recorded.

### Work flow

**Fig 1:**
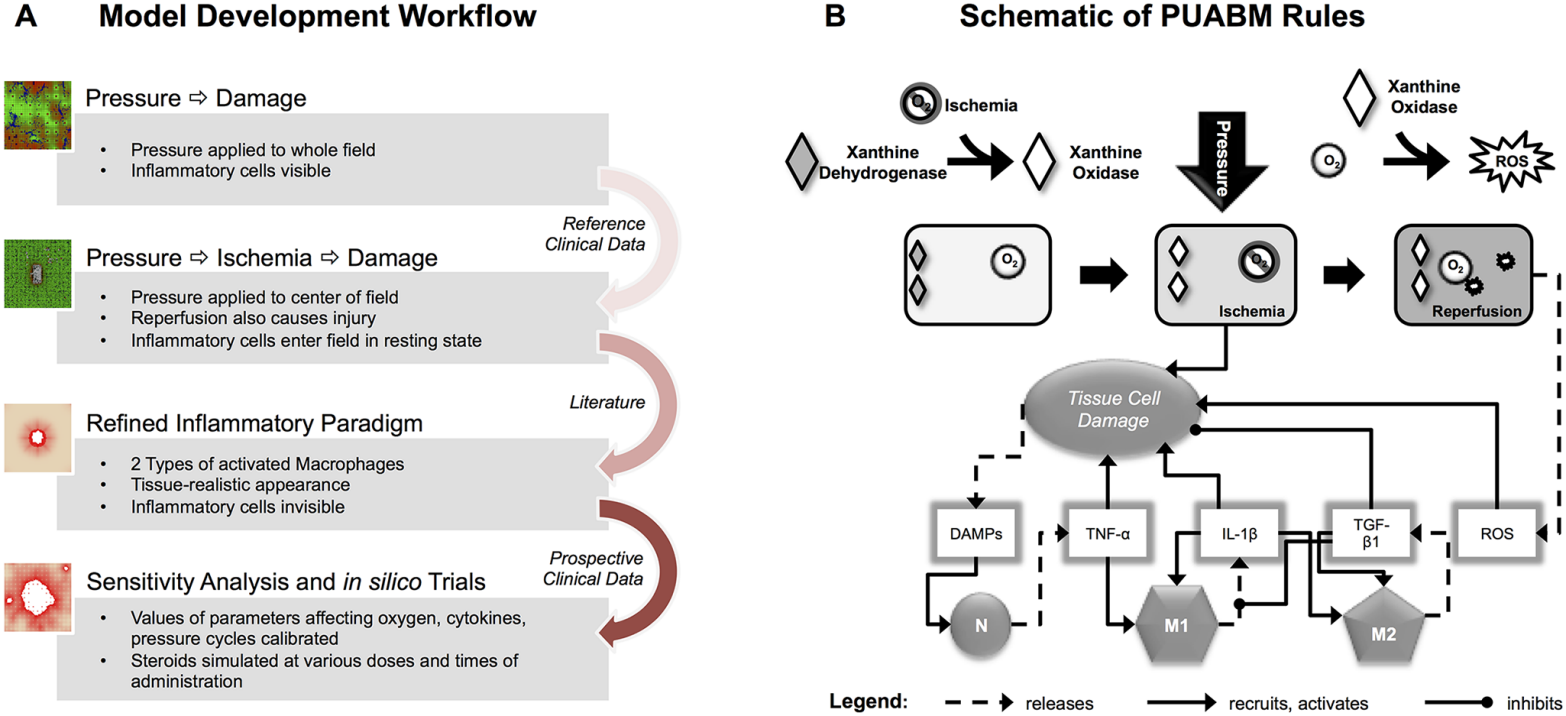
Work flow and schematic of the PUABM. (**A**) Work Flow utilized in constructing the PUABM. The ABM was built iteratively, incorporating domain knowledge and data from the literature, then validated with clinical data. Initially, pressure directly injured tissue, thereby inciting inflammation. Ischemia/reperfusion injury was next added as a cause of injury, and the complexity of the inflammatory response was increased. Clinical data were next used to calibrate parameters in the model, and the model was subjected to sensitivity analysis and *in silico* trials. (**B**) A schematic illustrates how model components interact to simulate two mechanisms of injury: ischemia/reperfusion injury and damage due to inflammation. A tissue cell (muscle, fat, or skin) is situated over a bony prominence. When pressure is applied, oxygen supply is reduced and the cell becomes ischemic, leading to tissue damage. Simultaneously, the enzyme xanthine dehydrogenase is converted into xanthine oxidase. Thus, reactive oxygen species (ROS) are produced when pressure is released and oxygen flows back to the cell, causing further damage. Tissue damage causes the cell to release DAMPs, which, along with local concentrations of pro-inflammatory cytokines activate both neutrophils (N) and macrophages (M1, M2) when these mediators are present above a given threshold parameter. For example, a sufficiently high local concentration of DAMPs activates neutrophils to secrete TNF-α, which can activate macrophages to a pro- inflammatory M1 phenotype. Tissue damage is ameliorated by anti-inflammatory mediators [TGF-β1].

The PUABM was built using an iterative approach (Fig 1A). Hereafter, when we use words or phrases that usually specify biological reality, such as “pressure,” “neutrophil,” “TNF-α,” “inflammatory mediators,” “cell,” “oxygen,” “ischemic,” etc. we are referring to PUABM components or generated phenomena unless clearly stated otherwise. A simpler model of pressure ulcer formation [22] was altered to increase mechanistic detail and create clinically relevant model output. Rational improvements were based on domain knowledge and data from the literature. First, the area of tissue simulated in the field was extended. Whereas in the previous version of the model pressure was applied evenly across the entire field, in this version, it was applied maximally (value determined by the parameter, *pressure-intensity*) to a circular area in the center of the field and decreasing radially outward. This allowed representation of pressure over a bony prominence and the surrounding tissue, which also experiences pressure, but to a lessening degree [23,24]. Next, the PUABM code was altered so that neutrophils and macrophages enter the tissue in a resting state and can be activated by one of two circulating mediators (TNF-α or TGF-β1). In the previous version of the model, all entering leukocytes were in the active state. After this modification, the activation state of neutrophils and monocytes/ macrophages was determined by the local concentrations of inflammatory mediators. These thresholds are explained further in the section below entitled, “Rules: Tissue Damage.”

In the previous version of the ABM, all cells and mediators appeared in a single visualization window. Epithelial cells ranged in color from green to red, depending on their level of health. Other cells were rendered in layers over the grid of epithelial cells[22,25]. In the PUABM presented herein, circulating inflammatory cells and mediators were each removed to a separate viewing window, allowing for comparison of spatio-temporal pattern of each individual component with any other. In the main window, stationary epithelial cells remained, but the colors indicating tissue health were altered to increase the realism: healthy tissue was now rendered as peach, mimicking the most common skin tones in the clinical cohort. Unhealthy tissue continued to be rendered as red, and cells that died disappeared from the grid, leaving behind a white empty space. Thus, each component of the model was represented according to the rules in the ABM, but by separating the viewing windows, we were able to view and compare spatial details of individual components (damage, mediators, etc.). Furthermore, we were able to compare epithelial damage patterns between simulations and clinical images, as described in later sections.

Mechanistic details in the PUABM were augmented by incorporating damage to tissue via ischemic injury and its counterpart, reperfusion injury. Damage directly resulting from pressure (as previously simulated [22,25]) was removed and tissue health was penalized for tissue cells receiving inadequate oxygen levels. (In the previous version, oxygen had positive effects on tissue health, but lack of oxygen was not damaging [22]). A mechanism based on the conversion of xanthine dehydrogenase to xanthine oxidase during ischemia [26,27] was also implemented. The accumulation of xanthine oxidase in ischemic cells represents the potential of a cell to experience reperfusion injury, due to formation of ROS when oxygen reperfuses [26]. The complexity of the inflammatory response was increased by creating two subpopulations of activated macrophages, one with pro-inflammatory (M1 macrophages) and another with anti-inflammatory (M2 macrophages) phenotype [28,29]. A fourth mediator was also added. Representing a canonical later-acting pro-inflammatory mediator, it is released by pro-inflammatory macrophages and labeled IL-1β.

After the mechanisms comprising the PUABM were set, model behaviors were explored over wide ranges of parameter values and default values were tweaked into ranges producing behavior that qualitatively matched the clinical data. Finally, we further investigated dynamics encoded in the model by simulating hypothetical and existing therapies. Corticosteroids were incorporated as a data layer that could be introduced to the tissue via blood vessels (as in an intravenous injection), while antibodies to DAMPs were simulated as a topical cream applied to the entire field. The dose and timing of administrations of each were varied to assess the viability of these treatment options.

### Model implementation

The PUABM was built using SPARK, a platform designed for agent-based modeling of biological systems, freely available for download at: www.pitt.edu/~cirm/spark [25,30,31]. SPARK models are written in a logo-like language called SPARK-PL and run on a java platform [32]. SPARK models contain Agents—autonomous entities that interact with each other and the environment, called Space—and Data Layers, corresponding to individual species in the environment that can diffuse, etc. The behavior of the model is determined by rules that govern how and when agents interact and react. These rules are generally written to be interpreted by one agent at a time, and therefore are necessarily restricted in scope (both time and space). A rule specifies how much an agent should move, produce, change an internal variable, etc. when it encounters a certain amount or type of data layer or agent in its immediate neighborhood. Model rules can be probabilistic in nature, allowing the model to evolve in a stochastic manner. Therefore, the behaviors and patterns produced by simulating several ticks (time steps) of the model in succession arise as emergent phenomena resulting from the accumulated actions of a population of agents over time. SPARK has several built-in standard methods that allow for convenient coding of common biological processes. For example, diffusion is encoded by the function *diffuse*, which implements a simple discrete approximation in which each data layer cell shares a given percentage of its value with its eight neighbors. Examples of other methods that were used in this model include *wiggle* and *jump* to approximate undirected random movement and *sniff*, for chemotaxis. See pseudocode (S1 Text) for all methods used.

The components of the PUABM are described in Table 1, and the operant biological mechanisms and tissue structures are shown graphically in Fig 1B. In the PUABM, simulation time is linked to actual time using the lifespans of cellular agents. A simulated macrophage lifespan ranges from 100–150 model time steps (ticks), corresponding to 100–150 hours (4–6 days) of real time. Neutrophil lifespans range from 10–20 ticks (hours) in the tissue, but increase when neutrophils become activated. Each simulation takes roughly five minutes to compute on a supercomputing node containing 32 process cores, each running at 4.7 GHz, making it possible to complete approximately 1000 simulations per day. The model contains 68 numerical parameters that are set by the modeler and 11 random variables (whose values are drawn from a corresponding uniform distribution when necessary during the course of a simulation). At any given tick, the maximum number of agents computed in the model is on the order of 10^5^.

**Table 1:** Summary of agents, data layers, and their interactions encoded in the ABM.

Substances in ABM	Cell Sources in ABM	Biological Functions in ABM
**DAMPs**	secreted by damaged Tissue cells	Chemo-attract and Activate Neutrophils
**TNF-α**	secreted by Activated Neutrophils	Chemo-attract resting and M1 Macrophages, activate Macrophages to M1 state
**IL-1β**	secreted by M1 Macrophages	Chemo-attract resting and M1 Macrophages, activate Macrophages to M1 state, at high levels activate Macrophages to M2 state
**TGF-β1**	secreted by M2 Macrophages	Chemo-attract resting and M2 Macrophages, activate Macrophages to M2 state
**ROS**	secreted by Tissue Cells on Reperfusion, by Neutrophils on death	Injure Tissue Cells
**Oxidase**	produced inside Tissue Cells during Ischemia	React with Oxygen to produce ROS
**Oxygen**	released by Blood Vessels	Necessary for Tissue Cell health, react with Oxidase to produce ROS

Substances are all encoded as Data Layers; Cells are all encoded as Agents, each with its own type. See Fig 1B for schematic.

#### Rules: Homeostasis/baseline architecture

The pseudocode in S1 Text details all model rules. In brief, the architecture of the PUABM is abstracted from healthy tissue. The PUABM consists of a layer of tissue cells fed by blood vessels throughout the tissue that carry oxygen and inflammatory cells. Instead of representing red blood cells explicitly, their presence is implicit since oxygen flows unaccompanied through blood vessels. Inflammatory cells arrive to the tissue in their native resting state and are not activated unless danger signals are released from damaged tissue. Resting inflammatory cells move randomly, but can be chemo-attracted to local mediators via the SPARK function, *sniff*.

The agents in the PUABM represent either cell types or cellular structures: neutrophils, macrophages, tissue cells, and blood vessels. Data layers are employed to represent mediators in a computationally efficient manner (e.g., diffusible cytokines, free radicals, oxygen, xanthine oxidase, and exogenously-administered drugs). As mentioned above, each tick (time step) of the model represents one hour of real time. On each tick, the agents behave according to rules specific to their type: they produce and consume oxygen and mediators, are chemo-attracted to and activated (undergo a state change) by mediators; and they die, according to their lifespans. Mediators, represented in data layers, can be consumed or produced by individual agents or in reactions with other mediators. They undergo diffusion and degradation at each tick.

To mimic natural patient-to-patient and cell-to-cell variability, some pseudo-random variables were built into the model. These values were drawn as necessary as simulations progressed, using the Mersenne twister (as implemented in the Colt library, http://acs.lbl.gov/ACSSoftware/colt/readme.html).

Some examples of random variables are size of each blood vessel agent (the blood vessels are all sized within a range but not exactly the same), and probabilities governing state changes of macrophages and neutrophils (a threshold must be met for the agent to be eligible for state change, but the random variable decides whether the transition actually happens). The function of the thresholds was to partition the interval (0,1). Accordingly, a uniform distribution was the basis of pseudo-random number generation. Similarly, blood vessels were assumed to be sized uniformly across a narrow range: the largest was less than twice the size of the smallest.

#### Rules: Pressure on/off

The turning of people with SCI while they are lying in bed was simulated as alternating cycles of pressure (on/off). In the PUABM, pressure is simulated by constriction of blood vessels, decreasing the amount of material that can flow through them. Without oxygen, tissue cell health in the PUABM begins to decline. Ischemic tissue cells increase xanthine oxidase enzyme activity, which represent the capacity of the cell to produce damaging free radicals upon reintroduction of oxygen (pressure turned off).

During a simulation, when pressure is released (turned off), blood vessel sizes increase, and oxygen again perfuses these cells, reactive oxygen species (ROS) are formed proportionally to the concentration of xanthine oxidase present in that cell. Free radicals cause damage to the immediate cell and those they encounter via diffusion, but they do so in a stepwise manner: tissue cells show no sign of damage from radicals until they have accumulated a certain threshold of insults. At that time, their health is reduced drastically.

#### Rules: Tissue damage

Each tissue cell in a simulation has an intrinsic variable called “life,” which ranges from 0 (dead) to 100 (perfect health). Tissue damage for a single cell is the opposite of life (100-life) and the measure for the whole field, total tissue damage, is simply the sum of each cell’s damage score. This number gives a general sense of how much injury has accrued throughout the course of a simulation.

$$Tissue\;Damage\;\left(total\right)\;=\;\underset{All\;Tissue\;Cells\left(i\right)}{\sum }100-life\left(i\right)$$

A cell’s life score is determined by local concentrations of oxygen, TNF-α, and TGF-β1. These represent the contributions of ischemia and inflammation to epithelial cell health. To mimic ischemic injury and recovery upon reperfusion, oxygen can be either beneficial or detrimental. We choose to model ischemia by comparing local concentrations of oxygen to a threshold, above which tissue health improved and below which ischemic damage was incurred. To mimic both damaging and pro-healing effects of inflammation, in the PUABM TNF-α decreases cell health while TGF-β1 supports healing [29]. Tissue cells that are stressed (have decreasing life scores) release diffusible danger signals (Damage-Associated Molecular Patterns [DAMPs]) that stimulate the inflammatory response by triggering the secretion of cytokines by inflammatory cells [33] Three diffusible mediators represent the canonical early pro-inflammatory response, the canonical slower pro-inflammatory response, and the canonical anti-inflammatory response, each of which is secreted by activated neutrophils or M1 or M2 macrophages. Neutrophils and macrophages are initially in a resting state, and are activated by local concentrations of mediators in a threshold-dependent manner. A local concentration of DAMPs above a certain value will activate nearby neutrophils to produce early pro-inflammatory mediators (abstracted as the pro-inflammatory cytokine TNF-α). At a certain threshold of local TNF-α concentration, resting macrophages will be activated to a M1 phenotype and begin secreting longer-acting pro-inflammatory mediators[34] (abstracted as IL-1β). TNF-α also causes damage to nearby tissue cells, thus re-stimulating the pro-inflammatory response. Local concentration of IL-1β above a threshold will activate macrophages to a M1 (pro-inflammatory) phenotype, and above a higher threshold, IL-1β will induce macrophages to a M2 (anti-inflammatory/reparative) phenotype[35]. These phenotype changes are reversible, meaning a M2 macrophage could be induced to switch to a M1 phenotype, depending on the local concentrations of mediators as outlined above (see Table 1 and pseudocode in S1 Text for rules governing macrophage state changes). Active M2 macrophages produce anti-inflammatory mediators (abstracted as TGF-β1), which above a threshold will cause further activation of M2 macrophages (Fig 1B). TGF-β1 also increases health of nearby tissue cells.

#### Model calibration

A default set of parameters was based on a model time step of 1 hour, which informed the lifespans of Neutrophils and Macrophages [28,29,33,34](see S1 Table for parameter values). Due to the lack of appropriate data, many parameter values were selected to achieve appropriate qualitative behavior at the cellular or molecular scales [36]. Accordingly, parameter values were modified one at a time until the model behavior aligned qualitatively with the rules to which they applied and baseline expected behaviors. The first test was to ensure that the “healthy” tissue was stable, i.e. the tissue was able to remain healthy when unperturbed. When damage appeared in simulations of undisturbed tissue after several thousand ticks (simulating nearly 7 months of real time), it was noted that oxygen levels at the edges of the grid were below the threshold at which tissue health began to decline, leading to tissue damage. Since unperturbed tissue should have plentiful oxygen and would not spontaneously degrade, diffusion rates were increased to ensure a more constant oxygen level across the grid. Similarly, the parameters controlling steroid inhibition of neutrophils and macrophages were tuned so that different doses of steroids produced a range of neutrophil or macrophage inhibition.

Other parameters were adjusted so that simulated ulcers evolved comparably to those in clinical images. Some properties that were compared included size of ulcers over time relative to first clinical observation or moment of ulceration in the model and extent of tissue damage in simulations or discoloration in clinical subjects beyond the outer edge of ulceration. For uniformity of appearance, only sacral ulcers within the clinical cohort were considered. As clinical images were used to define the baseline conditions of the PUABM, consequently this model is calibrated to the unique properties of SCI tissue (as we have suggested in a prior modeling study [25]). By altering a subset of the default parameters in the PUABM, the model could be calibrated to match time courses from a different cohort of patients or type of ulcer.

Several parameters represented lumped processes, and therefore it was necessary to make reasonable assumptions as to their values, accounting for relationships to other parameters. For example, TNF-α in the PUABM represents the collective actions of several pro-inflammatory species. Therefore, it would be inappropriate to base physical parameters (e.g. diffusion constants) on those known for TNF-α protein. Other parameters were tuned such that key behaviors of the PUABM were upheld. For example, in order to maintain reversibility of macrophage phenotypes, mediator thresholds of M1 and M2 activation were tuned so that neither population could too quickly or easily dominate without a significant and sustained source of the appropriate mediator. Certainly, many other default parameterizations could also have met these criteria, but they likely exist within ranges close to the values chosen in our parameterization. As explored later, model sensitivity analysis revealed several nearby regions of parameter space that yielded irrelevant or non-biological outcomes.

In examining and plotting the inflammatory dynamics resulting from the PUABM, some adjustments were necessary in order to bring the cell populations all into comparable ranges. Because the agents in the model are represented as unit-less numbers rather than specific numbers of cells, [36] absolute population sizes from the PUABM are meaningless. For a given cell type at time t, its population count was normalized by dividing by the Euclidean norm of the vector containing counts of that cell type for each tick throughout that simulation. This procedure allowed us to compare the relative timing of the peaks of each population. Presumably, it is not only the number of cells present that determines the level of the response, but also how sensitive those cells might be to their environment, and *vice versa*. Therefore, instead of calibrating for total number of cells, we focused on achieving reasonable qualitative behaviors in the tissue as a whole.

### Simulations of acute inflammation incited by initial injuries of varying degree

**Fig 2:**
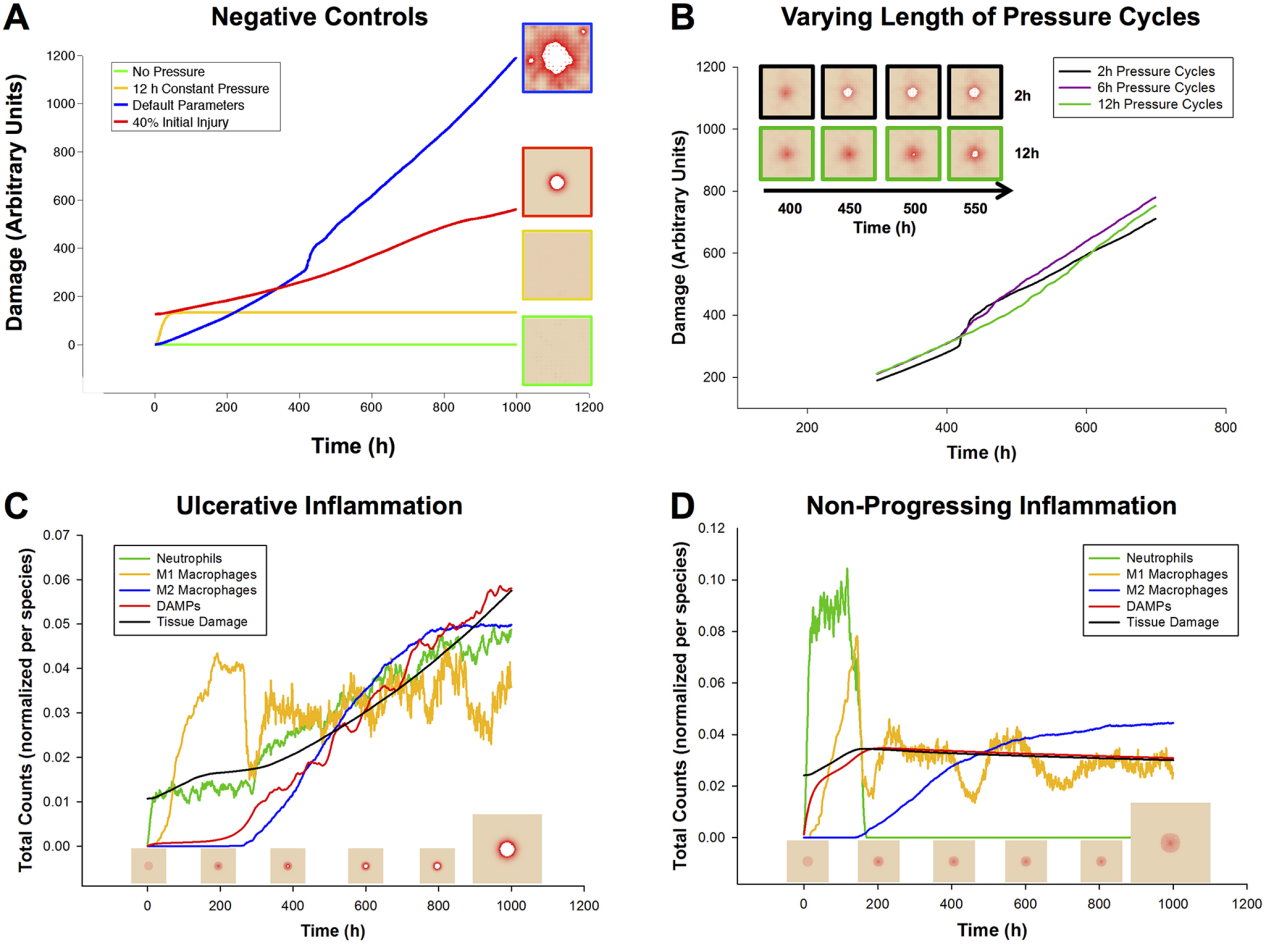
Model Verification: Mechanisms Lead to Expected Behaviors in Baseline Conditions. (**A**) Negative controls verify that the model behaves as expected in various situations. Green: undisturbed, tissue health is stable for >5000 hours (data past 1000 h not shown). Yellow: an initial period of 12 h of ischemia causes damage to the tissue, but after release no further damage was incurred. Red: a characteristic damage curve for an ulcer caused by acute inflammation after 40% initial injury (similar to 2C). Blue: a characteristic damage curve for a pressure ulcer resulting from the default parameters for the model. (**B**) The ischemia/reperfusion injury mechanism was validated by varying the period of pressure (on/off) cycles. Increasing the length of a pressure cycle allowed us to decrease the number of reperfusion events over the same length of ischemia. Pressure switched from on to off (or vice versa) once every 2, 6, or 12 h. y-axis is total damage, x-axis is time (h). Pressure cycle length did not seem to affect the total amount of damage until just after t = 400 h. At that time point, the simulations with the shortest cycles (black) show a sudden increase in damage, which visually corresponds to the formation of an ulcer (see inset). Eventually, ischemic injury in simulations with the longest cycle length (green) causes these simulations to incur more overall damage. (**C**) A 35% initial injury without pressure is sufficient to induce self-perpetuating, damaging inflammation, leading to an ulcer. The relative dynamics of the response are as expected from the literature: an initial influx and activation of neutrophils, followed by M1 macrophages, and then followed by M2 macrophages. (**D**) In 10–20% of simulations with the same parameters and starting conditions, though the inflammatory response was incited, it did not become self-sustaining and consequently no ulcer formed. The trajectories of inflammatory cells are characteristic of each of these outcomes. Data were normalized per cell type, and so quantities are not relative.

Pressure was a key factor in simulations of the PUABM. In simulations of the acute inflammatory response in the absence of pressure, as in Fig 2C and 2D, the differences in outcomes were most dramatic, as they either did or did not result in an ulcer. In contrast, when pressure was added, the differences in predicted outcome were more subtle: an ulcer always formed, but it was associated with varying degrees of overall damage. To better define the mechanisms influencing this bifurcated outcome, we initially carried out simulations of acute inflammation in the absence of pressure.

Acute inflammatory dynamics were incited by an initial injury to the center of the tissue. We first varied the intensity of the initial injury in order to determine over what range of injury both outcomes persisted. Because we found that the frequency of ulcerative inflammation was correlated to the intensity of initial injury, we focused on simulations with a 30% initial injury—the level at which 50% of simulations resolved and 50% formed an ulcer (see S2 Fig).

### Approximating damage distribution in simulations

We approximated the distributions of total tissue damage in PUABM simulations using a Gaussian Mixture Model fit using the Expectation-Maximization algorithm, as implemented in the Matlab function, *gmmfit*. We repeated this process varying the number of Gaussians in the model from 1 to 4, and then compared the goodness-of fit for each one, using Aikikae and Bayesian Information Criteria. This step allowed us to determine quantitatively the number of underlying Gaussian models that give rise to the pattern we see, an important step when the total damage from each outcome does not vary significantly. We selected the model that was a mixture of two independent Gaussian distributions because this model yielded the lowest scores for both Aikikae Information Criteria (AIC) and Bayesian Information Criteria (BIC), shown in S2 Table.

### 1-Nearest Neighbor analysis of simulation results

Following the work of Xing et al. [37], we employed a 1-nearest neighbor (1NN) approach to automatically segregate simulations ending in ulcers from those that displayed resolving inflammation. The training set consisted of data from 100 simulations, labeled according to which endpoint was reached (resolved or ulcerated). For each simulation in the test set, a pairwise distance was computed between itself and every simulation in the training set. The unlabeled test sequence was assigned the same class label as the training sequence that was closest in distance to it, its “nearest neighbor.”

This method relies heavily on the choice of distance metric, in this case the Euclidean distance between sequences. To take advantage of the time dependence of the features, we considered each simulation to be a sequence, wherein every entry in the sequence was a time point from a simulation. Each of those entries consisted of either a single value (e.g. total oxygen at tick t) or a 13-dimensional vector of model component values. For univariate time series, we calculated the Euclidean distance between each pair of vectors consisting of measurements of a single feature through time. For multivariate time series, we calculated the Euclidean distance between two feature vectors at each time step and took the Euclidean norm of those distances to be the distance between the two simulations. To equalize the contributions of all features, their values were normalized to fall into similar ranges before calculating distance.

### Sensitivity analysis of model parameters

We next sought to define the model parameters that most affected simulation outcomes in the PUABM. Because there are more than 50 free parameters in the model, it was impractical to examine the sensitivity of key model outputs to all of parameter space at once. Instead, modules of rules in the model that impacted tissue health were identified and used to create groups of parameters. Parameters within these groups were then prioritized according to the mathematical degree of their effect on the system. For example, a parameter that sets the value of an exponent produces a more dramatic effect than one that sets a scalar multiple. Therefore, the first parameters varied were those controlling threshold values. Simulations initially varied parameters over coarse-grained and then finer-grained threshold value ranges (100 simulations per parameter value). Total tissue damage at time t served as a quantitative output measure. From this analysis, a sensitivity index could be calculated for each parameter, taking the ratio of change in damage to change in threshold value.

A second level of sensitivity analysis was designed to examine the interplay between two potentially related parameter values. This analysis allowed for a direct comparison of the sensitivities of two parameters, and also revealed any secondary effects that occurred when the two parameters changed in a combinatorial way. Parameters were chosen from the same “damage module” in order to get a sense of the relationships between “sub-processes” in each module. Snapshots of the tissue layer in the model at a fixed time point served as the output in order to assess overall damage, presence and size of ulcer, and other qualitative features.

### *In silico* clinical trials

Treatments with corticosteroids or neutralizing anti-DAMP antibodies were simulated as *in silico* clinical trials. These trials consisted of sets of model simulations in which parameters controlling drug dose and timing and tissue response were varied, each independently.

Corticosteroid administration was simulated as an intravenous injection. Therefore, steroid molecules (implemented as a data layer) were introduced to the tissue via blood vessels (and were restricted when pressure was applied). The mechanism of steroid action was to kill inflammatory cells, regardless of their state (active/ resting), as illustrated in S6 Fig. When neutrophils were killed, additional ROS was released by the dying cell (see S1 Text).

Anti-DAMPs were simulated as a topical cream administration. A uniform layer of this molecule was introduced onto the field as a data layer at the tick specified by the parameter designating time of onset. The method of action of the antibodies was controlled by a quenching reaction, wherein local concentrations of DAMPs were reduced by an amount proportional to the smaller concentration of the two molecules present: antibody or DAMPs (see S1 Text for pseudocode and also S7 Fig).

## Results

### Model description and work flow

The PUABM represents vascularized soft tissue (skin, fat, muscle, or a composite thereof) overlaying a bony prominence. The central hypothesis of this model is that when this tissue is damaged (which can happen in a variety of ways), further complications can evolve, including inciting further tissue damage. See the *Materials and Methods* for a more detailed description of model rules. Pseudocode is provided in the online supplement (S1).

Epithelial cell health in the PUABM depends on the continuous flow of oxygen to the field of simulated tissue. Pressure—such as that created by shifting a person’s weight—is simulated as compressing the tissue and vasculature over a bony prominence, indirectly causing damage to the tissue by restricting the flow of oxygen to the tissue and secondarily by formation of oxygen species (ROS) upon reperfusion (when pressure is relieved). Injured tissue incites activation of inflammatory cells, which bring mediators to the field, some of which injure the tissue further. As simulated cycles of pressure on/off are repeated, these mechanisms are sufficient to generate pressure ulcers *in silico*. The molecular and cellular components of the model are enumerated in Table 1.

### Model verification with literature-derived data

#### Simulations of negative controls

After tuning model parameters so that we were studying behaviors in realistic physiological ranges (see *Materials and Methods*), there were a few specific mechanisms whose behavior we sought to verify. It was important to verify that the damage accumulated throughout a simulation is in fact due to the mechanisms of injury encoded in the model, and not to other spurious mechanisms. Unperturbed, the tissue remained “healthy” for thousands of simulated hours (Fig 2A “No Pressure” data shown up to 1000 h; this behavior remained unchanged up to 5000 h). Some damage was observed when tissue was subjected to 12 h of simulated ischemia (Fig 2A “12 h constant pressure”), but no additional injury to the tissue was observed after release of pressure. By comparison, starting with a 35% injury over a patch of tissue at time 0 h (Fig 2A “35% Initial Injury”) was sufficient to incite the inflammatory response, ultimately leading to PU formation. However, this injury was not as damaging as repeated cycles of ischemia and reperfusion carried out throughout the complete time course (Fig 2A “Default Parameters”).

#### Simulations of ischemia and reperfusion injury

If tissue damage in the model is caused both by ischemia and by reperfusion, then, all else being equal, a period of ischemia would be expected to cause less tissue damage than the same period of ischemia followed by reperfusion. This ischemia-reperfusion (I/R) damage hypothesis was verified by varying the length of pressure cycles (turning rate) in PUABM simulations while measuring total tissue damage (Fig 2B). As expected, both ischemia and reperfusion caused tissue damage. Holding the amount of ischemic time constant while increasing the number of reperfusion events led to earlier ulceration and a more dramatic increase in overall tissue damage upon ulceration. With lower turning frequencies (and thus longer ischemic periods), the amount of damage increased steadily until ulceration became an inevitable outcome, whereas with shorter ischemic periods followed by short recovery, the tissue was able to maintain more health until ROS levels crossed the injury threshold, leading to an ulcer (see S1 Fig). In both cases, the level of damage incurred long term reached similar levels (Fig 2B, inset). These *in silico* experiments were concordant with *in vivo* studies in which rats were subjected to varying amounts of ischemia followed by reperfusion injury [38]. As in our simulations, rats with more reperfusion events incurred more damage overall.

#### Simulations of acute inflammation yield two predominant outcomes

The rules governing inflammatory mechanisms in the PUABM are based on dynamics of acute inflammation. These dynamics were initially tested in a simulated acute wound, without repeated pressure cycles (Fig 2C and 2D). As each mediator in the simulation represents an amalgam of several mediators, the dynamics were validated at the cellular level. In a successful implementation of these mechanisms, we hypothesized that tracking the activation of neutrophils and macrophages would reveal cellular dynamics similar to those found in settings of acute inflammation.

**Fig 3:**
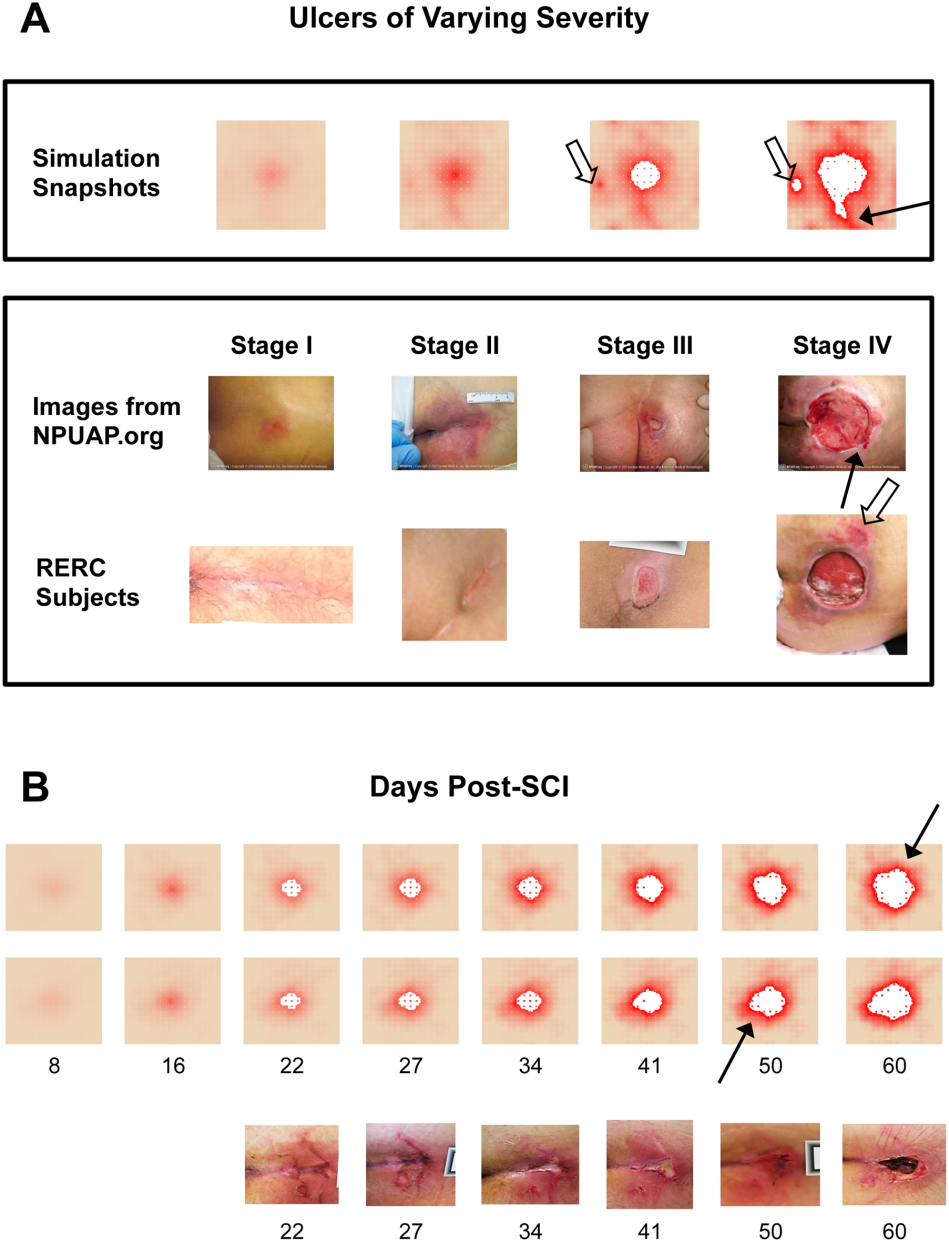
Simulations of the PUABM match key features of clinical images. (**A**) Simulations achieved visual appearances with characteristics similar to each stage of PU development. The first row of clinical images come from the National Pressure Ulcer Advisory Panel (images from npuap.org, used with permission) and are of different subjects. Images in the second row are from people with SCI enrolled in a prospective study of PU at each stage. Irregular shapes and increasing nearby damage are observed in both sets of clinical data. (**B**) Numbers indicate days post-injury. Simulated ulcers evolve with visual characteristics that match PU progression observed in people with SCI. Two simulation time courses are matched against one patient from our study. We match key features: irregular shapes, nearby satellite ulcers (open arrows), jagged edges (solid arrows), and decreasing tissue health across the field.

Interestingly, two characteristic patterns of inflammatory cell dynamics emerged spontaneously from the same set of default parameter values. We explore the potential causes of these two patterns below, but we initially sought to verify that both patterns agree with inflammatory dynamics reported in the literature. As observed in situations of self-sustaining inflammation [39], all cell counts in the first pattern reached a maximum and were still increasing at the end of the simulation (Fig 2C). Conversely, as one would expect to see in a resolving inflammation scenario [40], every cell type except M2 macrophages peaked well before the end of the simulations, displaying the second characteristic pattern (Fig 2D). In such simulations, neutrophil counts dropped to zero relatively early in the time course and did not rise again. Tissue cell counts from simulations corresponding to each of the characteristic patterns suggested that every simulation that had the first pattern of inflammatory dynamics resulted in an ulcer, while all simulations corresponding to the second pattern did not. This was also verified by examining visual outputs from simulations with both types of inflammatory dynamics (Fig 3).

### Model validation with clinical data

In simulations of the PUABM, tissue health begins to decline within the first few hours of pressure cycling, first in the area where pressure intensity is greatest, directly over a simulated bony prominence. This is manifested by a change in color from peach to red (similar to the erythematous appearance of inflamed tissue; notice the area of redness developing over time in the simulations [Fig 3B, top 2 rows)]. While all of the patients in our cohort who had detectable ulcers at this early stage had light skin tones (and therefore simulations did as well), model parameters could be adjusted to match darker skin tones. The simulated tissue is able to recover some health in the very first rounds of pressure, during periods of reperfusion, but after a certain point, the PUABM tissue field remains red (damaged) despite reflow of oxygen and leukocytes to the region. This is comparable to a region of un-blanchable skin erythema that is the diagnostic criteria for a Stage I PU [14]. Tissue health declines further as the simulation progresses, increasing the intensity and radius of redness. Eventually, tissue cells begin to die and exit the simulation, leaving behind a white patch to indicate lack of cellular activity at that position (see 4^th^ panel [day 25] of the top 2 rows of Fig 3B). The first instance of tissue cell death was considered to be the opening of a PU. From this time onward in the simulation, the PU was observed to grow outward while cells near the edge continued to decline in health.

Simulations of the PUABM were initially compared to reference clinical images of PU severity (see Fig 3A). The National Pressure Ulcer Advisory Panel (NPUAP) has issued guidelines classifying ulcer severity by depth [41], i.e. layer of tissue affected: skin, fat, muscle, or bone. The prevailing notion is that some ulcers begin as deep tissue injury before opening to the epidermal surface [42]. While the PUABM is two-dimensional, simulations were nonetheless able to capture the appearance of a variety of PU of various degrees of severity. As shown in Fig 3A, simulations achieved appearances similar to all four stages of ulcer progression. Specifically, the pattern of damaged tissue surrounding the ulcers was similar in simulations and clinical images. In both simulations and clinical ulcers, tissue immediately surrounding the ulcer showed the most damage, while tissue farther away from the pressure point generally appeared healthier (though not without some smaller areas of damage).

The simulations also recapitulated evolution of PU development observed in a prospective patient cohort of 49 patients (see *Materials and Methods*). The average time to ulceration post-injury was 20 ± 12 days among the RERC subjects. With default parameters in the PUABM, the time to ulceration was 405 ± 6 hours (17 ± 1 days); there was no statistically significant difference between the simulation predictions and the actual RERC ulceration times (p = 0.606 by Mann-Whitney comparison on ranks). We take this agreement to be one measure of validation of the mechanisms encoded in the PUABM and their associated parameter values. The timing of ulceration in the PUABM was not encoded explicitly; in fact, ulceration itself was not encoded in the model. Rather, the progression to ulcer occurred as a result of the accumulation of many smaller tissue-damaging events in a larger context (field of tissue). The parameter values that allowed this progression to agree with clinical data do not govern ulceration itself, but only control these smaller events (e.g. macrophage lifespan, cytokine secretion rates, etc.). When taken together across the whole simulation field, we conclude that PUABM-generated patterns are comparable to images of tissue in human patients.

The reference clinical images are static snapshots of a dynamic process; moreover, the images are from different individuals. Accordingly, individual simulations of the PUABM were compared to the dynamics of PU formation in a single individual. Note that clinical days are approximate, and that we sought to determine if the PUABM would result in qualitative concordance with clinical images. Therefore, we allowed for alignment of simulation images to clinical snapshots within a several-day window, especially early in the process of ulcer formation (when it is quite difficult for caregivers to define nascent ulcers). Fig 3B shows snapshots from a single simulation in which PU reached visual appearances with striking similarities to time courses of individual patients. Key characteristics of the PU arise in both simulations and patients. Though the simulated bony prominence is circular while in reality the sacral pressure area is approximately a triangle, both simulated and actual patients develop ulcers with irregular shapes. This is especially noticeable from day 33–34 onward in Fig 3B, for both simulations and clinical images. Furthermore, in both simulations and clinical subjects, once an ulcer formed, a second nearby ulcer was more likely to develop. Secondary ulcers are marked with open arrows; they are apparent in RERC subject showing a stage IV ulcer and in the last simulation panel of Fig 3A, though the ulcer also appears in the third simulation panel in a less severe state. In our simulations, decreased oxygen and increased tissue damage surrounding the primary ulcer contribute to weakened surrounding tissue that is more vulnerable to ulceration than tissue that is farther away. The model also recapitulated irregularly shaped ulcer boundaries: compare the last panel of Fig 3A and both rows of Fig 3B to images from stage IV NPUAP and the clinical time course in Fig 3B. Jagged edges were also noted in both simulations and clinical images, which are indicated with solid arrows and appear in the rightmost panels of Fig 3A and across time courses in Fig 3B.

### Nearest neighbor analysis suggests that pro-inflammatory mediators drive tissue damage in the PUABM

In order to gain insights that may at some point be of diagnostic utility, we sought to understand the range of behaviors that the PUABM can achieve, as well as the conditions that accompany these behaviors. To inspire new treatment strategies, we investigated the likely mechanism by which those outcomes are reached. While the structure of a given computational model can, of course, dictate its ultimate behaviors, much of the behavior of a model can be determined by the values of its parameters [43–49]. Accordingly, model parameters (including initial conditions, rates, thresholds, etc.) were varied alone and in combination with each other in order to gain an understanding of how variations in parameters relate to variations in output (time-evolving health of tissue). Tissue health was quantified by assessing overall damage as indicated by the life score attributed to each tissue cell, as detailed in the *Materials and Methods*. A second metric for tissue health was presence or absence and size of an ulcer, which was marked by the death of the first tissue cell. Taken together, total tissue damage and presence/ size of an ulcer allowed us to compare one simulation to another and qualify whether the outcome of one was better or worse than the other.

#### Bimodal behavior in simulations with pressure cycles

**Fig 4:**
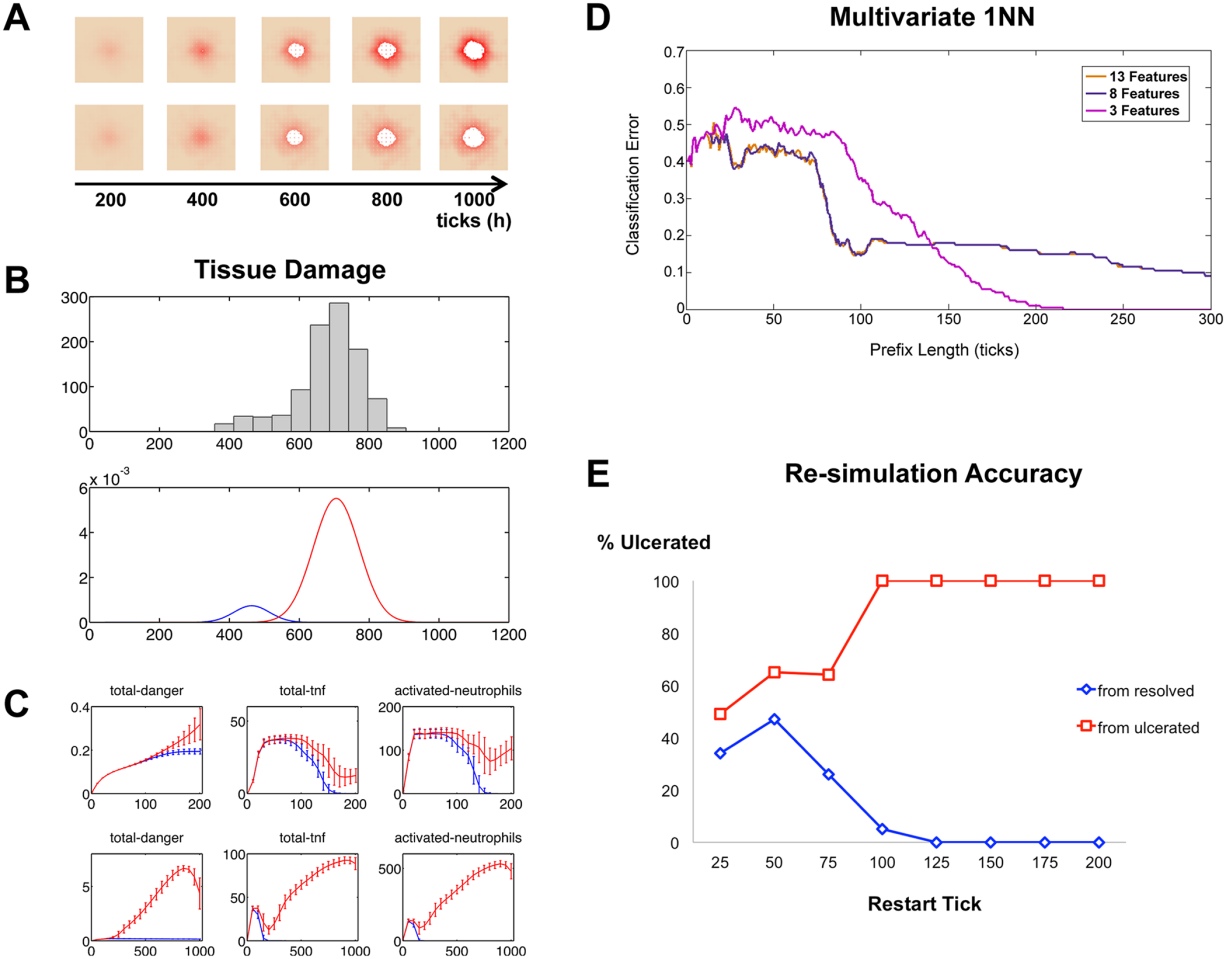
Bimodal outcomes are determined by 125 ticks (5 simulated days) prior to the appearance of post-SCI ulcers. Panel (**A**) shows two simulations that were initiated with the same set of parameter values but evolved different levels of damage over time. In panel (**B**) these levels are characterized by a bimodal distribution of tissue damage at tick t = 1000 (day 42). The upper panel is a histogram of these values and the lower panel shows the bimodal distribution estimated from 1000 simulations with the same parameters and initial conditions. (**C**) Some individual features display very different trajectories between resolved and ulcerated simulation outcomes. Means and standard deviations of 1000 simulations are plotted. Upper 3 panels are the features plotted over the first 200 ticks (8.3 days); lower panels show the full time course of 1000 ticks. (**D**) 1-Nearest Neighbor performance versus length of sequences in training set was determined for various numbers of included features. (**E**) Percent of simulations resulting in ulceration after restarting from saved states at indicated restart tick. The saved states were derived from simulations that originally resulted in either ulceration or resolving inflammation.

We observed that simulations could reach two distinct endpoints, even when initiated with the exact same parameter values. This could be a significant feature of the model, as a similar dichotomy is also observed in the clinic: two patients with otherwise similar health may have drastically different responses to the same apparent injury and similar subsequent treatment [50]. An understanding of how the model could arrive stochastically at two outcomes might lead to information that could help a clinician predict which patients might be headed for which outcomes. The dichotomous model behavior was most prominent in simulations of the acute inflammatory response in the absence of pressure, as in Fig 2C and 2D (see also S4 Fig), but was also observed when pressure cycles were applied (Fig 4A). With pressure applied, and other parameters set to default ranges, all simulations led to an ulcer; however, the amount of damage to the tissue surrounding the ulcer differed from simulation to simulation. Reducing this damage would be a desirable clinical outcome, because such damage reduction may prevent the worsening of that ulcer and/or lower the risk of developing secondary ulcers in nearby tissue. Histograms of total damage at t = 1000 from 500 simulations illustrate the complexity of this problem (Fig 4B, grey bars). Using the Expectation-Maximum algorithm, we found that the data were best approximated by a mixture of two Gaussian models, which suggested that there were in fact two levels of damage among the simulation outputs. In Fig 4B, the peaks estimated by the mixture of two Gaussians model are plotted below the tissue damage histogram for visual comparison. We notice substantial overlap between the two outcomes, and the mixing proportions of these two distributions estimated by the GMM fit are unequal. Using this parameterization of the model, the lower damage outcomes occur in only approximately 10% of the simulations. As with bimodal inflammatory responses to an acute initial injury, this mixing proportion could be tuned by using a different parameterization. However, it is difficult to calibrate this number to clinical outcomes because ulcers that resolve before they open at the surface often go unnoticed [51,52].

#### Pro-inflammatory mediators produced during simulations correlate with bimodal tissue damage

Once we verified that the bimodal behavior was quantifiable, we aimed to define the variables in the PUABM most predictive of the amount of damage. Accordingly, we examined both visual and statistical patterns in model components (e.g. each cell type and mediator). Using counts of each component as a time-dependent readout, we determined which of these features was most predictive of a particular level of overall damage. At each tick, recordings were made of total amounts of tissue damage, each mediator, oxygen, and counts of each type of activated cell. Analysis of these data suggested that a few features have patterns that differentiate between ulcerated and resolved simulations early in the time course (see S4 Fig). Certain features of simulation outputs *activated-neutrophils*, *total-tnf*, and *total-danger* appeared to differentiate between outcomes, especially between 100 and 200 ticks (Fig 4C). In contrast, several other features (*total-oxygen*, *blood-flow*, *total-antiox*, *total-radical*, and *total-oxidase*) did not appear to differentiate between endpoints.

#### Re-simulations starting as early as day 5 post-SCI consistently match original simulation outcomes

We next sought to find a mathematical description of the decision boundary between simulations that results in higher vs. lower amounts of tissue damage. The features that figured most prominently into this description, we reasoned, would be the ones that are most predictive of damage levels. Furthermore, we expected to find good prediction accuracy with sequences the length of a full simulation (1000 ticks, or 1000 simulated hours—approximately 5 weeks). We were not only interested in prediction accuracy, but also the “minimum prediction length” or the time point before which accuracy begins to decrease. We used the minimum prediction length as an indicator of the time by which the simulation was committed to one outcome or another. The first step was to assess the predictive power of each feature individually. The classification error rate of the 1-Nearest Neighbor Classifier trained on various subsets of the features for the first 200 ticks (8.3 days) or 50 tick (2 day) prefixes is shown in S5 Fig. While classification error ranged from above 60% to just below 40%, the error never stabilized, and no single feature achieved an acceptably low error rate.

We next repeated the classification, incorporating information from multiple features into each test and training sequence (a multivariate sequence). We assessed the performance of 1NN using various combinations of features: all 13 features, 8 features that were not inert across the first 200 ticks (all but *total-oxygen*, *blood flow*, *total-antiox*, *total-radical*, *total-oxidase*), and three features that appeared to differ the most among the two outcomes before 200 ticks (*activated-neutrophils*, *total-tnf*, *total-danger* (Fig 4C)). We found the classifier performed best when trained on a parsimonious set of three features. While removing inert features did not appear to improve performance (Fig 4D), pruning to only 3 features did result in improved performance (Fig 4D). A possible explanation for this finding is that the remaining 5 non-inert features contributed noise rather than information that was correlated with outcomes. We expected that in this last case we would achieve a perfect classification (0% classification error) because there was complete separation between the two peaks. By 200 ticks (equal to 200 hours or roughly 8.3 days of real time), we had in fact achieved 0% error rate using 1NN with *total-tnf*, *total-danger*, and *activated-neutrophils* (Fig 4D).

While 1NN using 3 features was able to separate perfectly low damage from high damage outcomes by 200 ticks (i.e. 8.3 days post-SCI), it is possible that final damage levels were determined even earlier in the simulations. We next sought to examine whether 1NN truly identified the decision time point in the model. To do so, we asked if restarting a simulation from early time points but with a different random seed would lead to the same outcome as the original simulation. For ten simulations (five resulting in ulcers and five resolving), we recorded all state variables at intervals of 25 ticks (approximately 1 day) until t = 200. Starting from each set of saved states, we initiated 20 simulations and carried them out for 500 additional ticks, or about 20 simulated days. This process yielded 100 simulations from each of eight starting time points that were originated with states from resolving simulations, as well as another 100 simulations starting from each time point in ulcerating simulations. The ulceration status of each of these new simulations was noted, and the rate of disagreement with the source simulations was plotted for each starting time point (see Fig 4E). Contrary to the results from 1NN, the simulations seem to be determined before t = 125. In all but one source simulation, all re-simulation outcomes matched original simulation outcomes by t = 100. These findings imply that there are model configurations determining PU fate from ~4 days post-SCI. It would be worthwhile to discover these exact configurations to determine whether they might be useful in a clinical setting.

### Sensitivity analysis

#### Global sensitivity analysis of the PUABM suggests that inflammation and oxygen determine to tissue damage, but reperfusion is critical to ulceration

The goal of a global sensitivity analysis is to understand how changing model parameter values affect the outcomes produced in simulations. Others have used sensitivity analysis techniques to analytically determine which parameters were most highly associated with metrics of model outcomes [43,49,53]. This technique is particularly useful to determine which parameters of a model are most influential toward model outcomes. Because many of our parameters represent lumped estimates or qualitative relationships, and since we were interested in which of only three high-order mechanisms were most important, we hypothesized that we would be able to parse out parameters controlling such high order information without performing an exhaustive sweep of such a large parameter space. We first sought to explore how various model parameters might interact with each other to cause complex tissue damage outcomes. We performed a sensitivity analysis aimed at observing the relative and additive effects of each source of damage.

**Fig 5:**
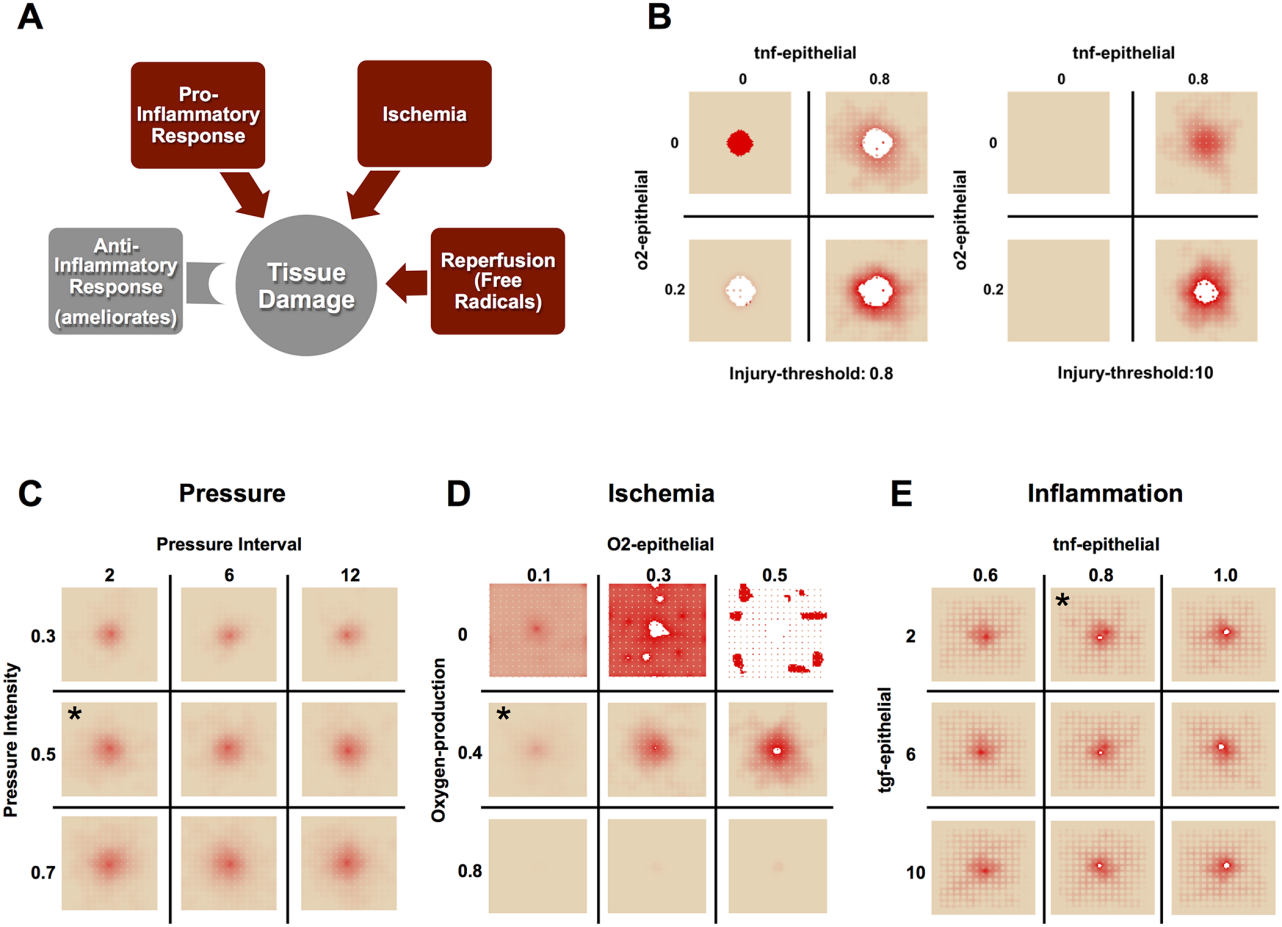
Sensitivity analysis reveals a unique contribution for all damage mechanisms, but simulated tissue is most sensitive to oxygen. (**A**) We partitioned parameter values according to which damage mechanism they affected. Panel (**B**) shows a global sensitivity analysis comparing the effects of the three mechanisms of damage on tissue health outcomes. Each mechanisms is simulated at two levels: with maximal or negligible effects. In panels C-E, sensitivity analysis was performed on parameters within each mechanistic module. All sensitivity analyses are shown with model snapshots at time t = 400 h (approximately 2.5 weeks). Default parameters are marked with asterisks. (**C**) Tissue sensitivity to oxygen parameters. The parameter governing oxygen production varies in each row. Each column represents a value of the parameter controlling how sensitive the tissue is to local oxygen concentrations. When oxygen production is plentiful, the simulated tissue becomes insensitive to other oxygen parameters (bottom row). Panel (**D**) illustrates tissue sensitivity to pressure intensity and period. Pressure intensity varies in each row. Each column represents a value of pressure cycle length. As the cycle length increases, the number of reperfusion events decreases for the same period of ischemia. Increased pressure leads to more damage, while fewer reperfusion events lead to lower damage at t = 400 h. Panel (**E**) illustrates tissue sensitivity to inflammatory mediators. Each column represents a value of the parameter controlling how sensitive the tissue is to local TNF-α concentrations. The parameter controlling tissue sensitivity to TGF-β1 varies in each row. Increasing sensitivity to TNF-α leads to earlier ulceration and more damage, while increased sensitivity to TGF-β1 leads to decreased tissue damage.

The model includes three sources of tissue damage: lack of oxygen, presence of TNF-α, and presence of ROS (Fig 5A). TGF-β1 and IL-1β were not included in this analysis because they do not contribute to tissue damage directly. Thus, TNF-α, ROS, and oxygen are the three broad mechanistic sources of damage, and for each there exists a single broadly effective parameter that controls the magnitude of the effect of a given parameter on a simulation. Biologically, we view these parameters as representing an individual’s overall sensitivity to each of the three damage-causing species. The first step in this analysis employed two values for each parameter, corresponding to low (zero-effect) and high (default) levels (Fig 5B). The zero-effect level of each parameter was defined as the value at which that source of damage no longer contributed to a simulation (an individual would be completely insensitive to presence or absence of that species). PUABM simulations in which each of three broadly effective parameters was set to either default (definitely has an effect) or zero-effect values show explicitly how each of the sources of damage affects outcomes on its own and in combination with other damage sources (Fig 5B). We note that when the tissue is not sensitive to oxygen (left hand column of each panel), no ancillary damage accumulates even when an ulcer appears by t = 400 h. We chose this time point because differences between parameter sets were well pronounced by this time point. Sensitivity assessed at later time points proved to be less informative, as in these extreme conditions the ulcers had already progressed significantly. In several parameterizations, time points prior to t = 400 were also less informative, due to sudden ulceration in otherwise healthy-looking tissue.

The differences between simulations with and without sensitivity to oxygen (top row versus bottom row) are that of tissue damage severity. In all but one column, the bottom simulation (oxygen-sensitive) appears as a more severe version of the upper simulation (oxygen-insensitive). From this analysis, we hypothesize that while oxygen sensitivity contributes to tissue damage, it is not sufficient to cause significant damage or a PU, and furthermore we hypothesize that the lower the threshold of reperfusion injury, the more likely a PU is to form. Comparison of analogous simulations in each of the two panels (left panel is sensitive to ROS, right panel is ROS-insensitive) shows that often it is sensitivity to ROS that is the difference between ulcerated and damaged (but non-ulcerated) tissue in the PUABM. The simulation in the bottom right corner of the second panel shows a PU, but it is smaller and less extensive than the ulcer in the bottom right simulation of the left panel. This snapshot represents the type of PU that forms when parameters dictate that tissue is sensitive to all three damage sources. Based on these analyses, we hypothesize that tissue damage can be ascribed to inflammation, ulceration can be ascribed to ROS, and oxygen can exacerbate damage but may not be sufficient to cause significant tissue damage.

#### Module-based exploration of the PUABM suggests that tissue is sensitive to oxygen and pro-inflammatory mediators

We next sought to determine the degree to which parameters within each of the three mechanisms described above contribute to tissue damage. Within each mechanism, we identified parameters that were likely to have the greatest effect due to their mathematical form; i.e. thresholds were more likely to significantly influence outcomes than linear coefficients. Because injury in the PUABM is initiated by repeated pressure cycles, we first examined how sensitivity to pressure affected damage outcomes. To do this, we varied the maximum intensity of pressure (applied to the center of the tissue) and the length of the pressure interval (Fig 5C). This number indicates for how many consecutive ticks (simulated hours) pressure is applied, equal to the number of ticks during which pressure is removed. At t = 400 h (16 days) of simulated time post-SCI, very little effect of pressure interval length was observed. However, a slightly lower degree of damage was discerned in the medium-length pressure interval. Pressure intensity appeared to affect tissue health negatively.

Pressure influences tissue oxygen in the PUABM. The impact of oxygen on tissue health was therefore investigated by varying in tandem parameters controlling oxygen production (how much oxygen is released from a blood vessel at each time interval) and tissue sensitivity to oxygen (a scalar factor determining the degree to which epithelial cell health is impacted by local oxygen concentrations, same as in global analysis above) (Fig 5D). This analysis suggested that when oxygen production from blood vessels in the PUABM is low (or zero), tissue health is affected severely, but in a manner that scales with the parameter that specifies sensitivity to oxygen. At high oxygen production levels, however, there was no apparent sensitivity of simulated tissue health to oxygen concentration.

Simulated tissue sensitivities to pro- vs. anti-inflammatory/ pro-healing mediators were compared to each other (Fig 5E). This analysis involved comparing the effects of varying the parameter controlling the model’s sensitivity to TGF-β1 (the canonical anti-inflammatory/ pro-healing mediator) and the parameter controlling sensitivity to TNF-α (the canonical early-acting pro-inflammatory mediator). Although all simulation snapshots are taken at the same time point, PU appear by t = 400 h (16 d) with higher sensitivity to TNF-α; this does not occur at lower sensitivity to TNF-α. The effect of TGF-β1 on tissue health in the PUABM is less pronounced, though there is overall less damage as the sensitivity to TGF-β1 increases.

We examined some of the more detailed mechanisms contributing to the severity of inflammatory response by determining which of thresholds within that module were the most sensitive. The inflammatory response, like most biological processes, is regulated in part through signaling mechanisms that, in essence, define thresholds of activation [54]. Accordingly, various thresholds in the PUABM control state changes of inflammatory cells (e.g., when local TNF-α concentration is above threshold, macrophages convert to M1 phenotype.) There is a different threshold for each state change, as illustrated in Table 1 and Fig 1B. Sensitivity analysis indicated that the most sensitive threshold was the concentration of DAMPs necessary to activate neutrophils. We found that most thresholds had a small range over which the output was sensitive to the threshold value, and outside of this range the dynamics were stable. This was not true for thresholds controlling activation of M2 macrophages. For both IL-1β and TGF-β1, increasing the concentration necessary to induce M2 activation led to increased simulated tissue damage.

### *In silico* clinical trials for post-SCI pressure ulcers

#### Corticosteroids reduce overall tissue damage, but not post-SCI ulceration in simulations

**Fig 6:**
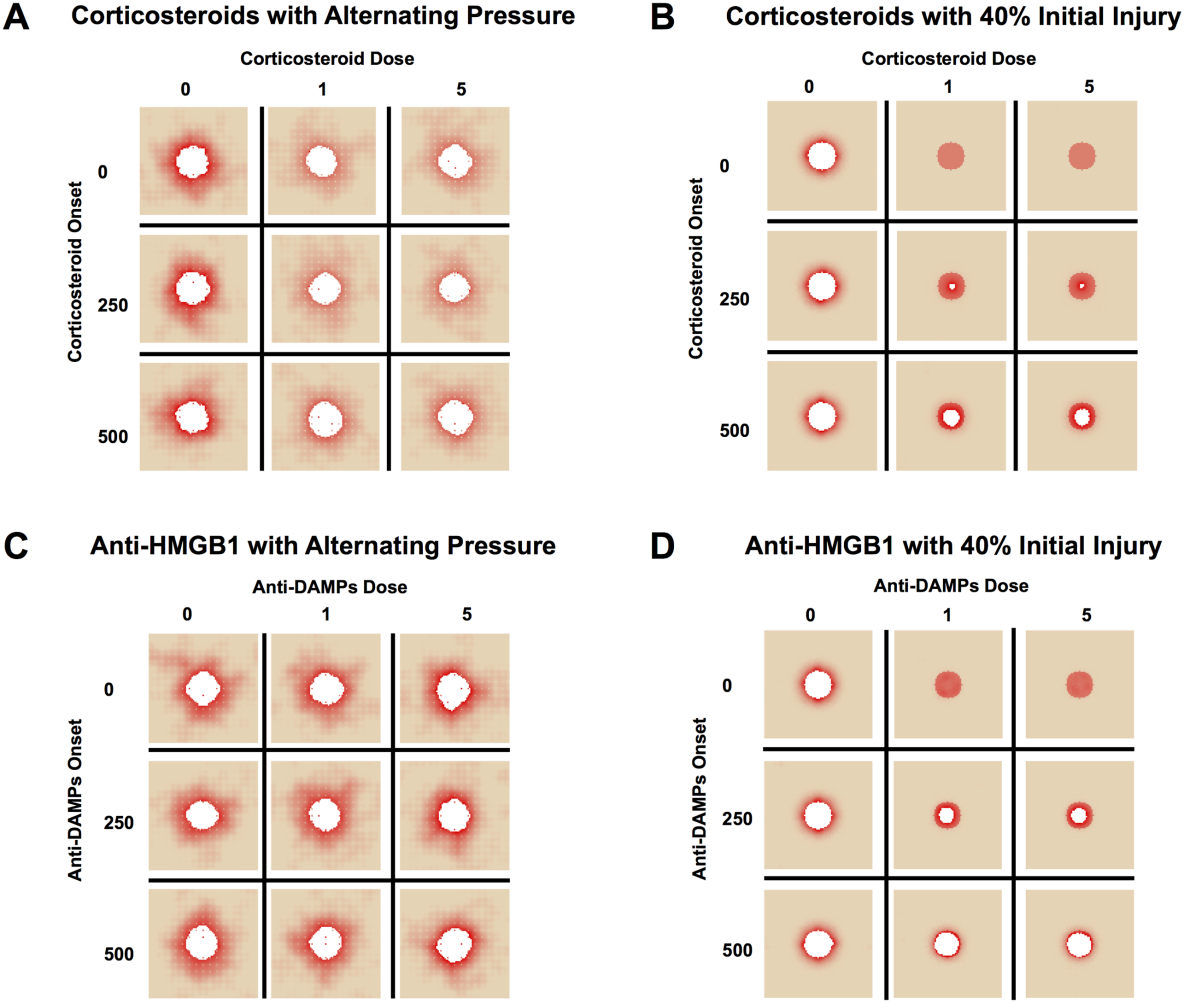
*In silico* clinical trials suggest little efficacy for corticosteroids or DAMP inhibitors. Simulations are shown at t = 700 h. We varied both the dose and timing of corticosteroid administration, simulated as an injection into the bloodstream, under (**A**) alternating pressure and (**B**) 40% initial injury conditions. When inflammatory cells were neutralized early enough but pressure continued, overall damage decreased, but ulceration was not prevented. Without continuous pressure cycles, the earliest dose of steroids was successful in stemming ulcer formation, but later applications did not. We then varied both the dose and timing of administration of a neutralizing antibody to HMGB1, simulated as a topical cream applied to the entire field. This targeted approach had (**C**) no apparent effect during simulations with alternating pressure, but (**D**) was able to slow ulcer formation after a 40% initial injury without pressure.

*In silico* (simulated) clinical trials are an inexpensive and increasingly popular means of gleaning translational knowledge from computational models [55–59]. Accordingly, these methods were utilized to test both current and hypothetical or cutting-edge therapies for inflammation in the setting of post-SCI PU. The feasibility of corticosteroids as a treatment was examined for this indication using the PUABM, varying dose and timing of corticosteroids to investigate whether reducing inflammation could delay time of ulceration and/or lead to less tissue damage (Fig 6). Corticosteroid administration was simulated as suppressing all inflammatory mechanisms. This effect was implemented by the following rule: any activated macrophages or neutrophils die upon encountering at least a threshold amount of steroid in their local vicinity of steroid (S6 Fig). We note that doses do not correlate directly to clinical doses, but instead to degree of functionality. For example, a simulated high dose (5) of corticosteroids shuts down macrophage and neutrophil activity completely, whereas lower doses all some cells to live and takes longer to see an effect. In simulations where corticosteroids were administered at the highest dose and before 250 hours, total damage was reduced. However, with simulated pressure cycling, PU still formed, suggesting that the lack of inflammation driven by macrophages and neutrophils was insufficient to ameliorate the damaging effects of ischemia and reperfusion (Fig 6A). By comparison, simulations with an initial acute injury but without pressure responded more favorably to steroid intervention (Fig 6B). These simulation results agree with the global sensitivity analysis of Fig 5B. It appeared that all doses of corticosteroids greater than zero were sufficient to achieve the full effect in both cases in the PUABM. There was, however, more damage incurred when corticosteroids were introduced at later times. While overall damage in simulations in which corticosteroids were introduced late was still less than simulations without corticosteroids, timeliness of corticosteroid application was anti-correlated with tissue damage incurred. This is likely related to the self-perpetuating nature of the pro-inflammatory mediators in this model. We hypothesize that early interruption of the positive feedback cycle was more effective in reducing inflammatory activity than the same amount of treatment later.

#### Anti-DAMP antibodies have no effect on post-SCI ulceration in simulations

DAMPs such as HMGB1 have become leading therapeutic targets for inflammatory indications [60,61]. Accordingly, dose and timing of anti-DAMP administration were varied over wide ranges using the PUABM (implemented as illustrated in S7 Fig); however, no apparent effect was observed (Fig 6C). This lack of efficacy in these simulations is perhaps unsurprising, as there are multiple methods of inciting inflammation encoded in the model. Similarly, in humans and experimental animals [33] there are several ways by which the body is alerted to cell damage and potentially harmful invaders. In the PUABM, inflammation is incited secondarily when tissue cells that have accumulated damage above a threshold can activate neutrophils directly, thus compensating for the lack of DAMPs. As with corticosteroids, the same treatment was simulated on an initial acute injury without pressure cycles, yielding similar results (Fig 6D).

## Discussion

We have previously put forward an approach involving extensive simulations validated against highly focused clinical data in order to streamline subsequent *in vitro*, *in vivo*, and clinical studies [62]. Herein, we demonstrate how a tissue-realistic ABM of ischemia/reperfusion injury and inflammation in epithelial tissue could recapitulate morphological features of pressure ulcers in individuals with spinal cord injury, in a manner that is highly consistent with clinical images. At the molecular level, the model was calibrated to *in vivo* studies of ischemia/ reperfusion injury in rat epidermis [38] as well as inflammatory dynamics reported in the literature. The model output matched clinical ulcers qualitatively, generating irregular shapes and jagged edges, as well as distinct, secondary foci of inflammation that could progress to ulcers, despite initial conditions simulating a circular area of bony protrusion. In further general agreement with clinical data, model simulations spontaneously reached endpoints of either pathogenic or resolving disease. Interrogation of this stochastic phenomenon *in silico* revealed that ulceration outcomes were determined before the appearance of an open ulcer—suggesting that early diagnosis and intervention are critical. From a diagnostic standpoint, our simulations suggest that the most important predictors of ulcer formation are tissue oxygen levels and the levels of pro-inflammatory mediators. Predictions of the PUABM could be compared visually with easily-obtained images of patient skin; thus, we suggest that with further calibration and validation, this model could eventually be used as a diagnostic aid to determine which patients are at higher risk for ulcer formation before ulcers progress beyond stage I. From the standpoint of therapeutic interventions, *in silico* clinical trials using the PUABM suggested that after a relatively early point in time, inflammation-targeting intervention are unlikely to prevent ulceration or reduce tissue damage. In our simulations, corticosteroids were incapable of preventing ulcer formation, though when applied early enough could reduce the amount of tissue damage surrounding the wound. Sensitivity analysis revealed that ulceration in the model was correlated to tissue sensitivity to oxygen. Thus, wound oxygenation may be a potential therapeutic avenue for post-SCI PU. To date, studies of hyperbaric oxygen treatment have not proven successful in treating PU [63]. This failure may be explained by the timing issues mentioned above; we hypothesize that oxygenation is key to prevent ulcers from forming, but less helpful once they do.

Where data were available, model parameters were calibrated to cell-level phenomena (e.g. lifespans). Otherwise, values were chosen that would yield tissue phenotypes relevant to healthy conditions. We reasoned that for any insights derived from the PUABM about ulceration to be meaningful, the same parameterization should allow tissue to remain healthy when unperturbed by pressure. For example, diffusion rates were adjusted to ensure adequate oxygenation to tissue that was not experiencing pressure. Diffusion represents an important link between spatial and temporal scales of the model, since diffusion governs the radius of effect a mediator could have. In early tests of negative controls, after several hundred hours of simulations with no pressure, we noticed tissue health declining at the edges of the simulation grid despite toroidal boundary conditions. We concluded that oxygen concentrations over time were not sufficient to maintain tissue health in those regions, and therefore our estimate of the diffusion rate was likely too low. By changing this parameter, tissue health was maintained when unperturbed for thousands of simulated hours. Importantly, other mediator diffusion parameters were also adjusted, which did not change the dynamics of the model.

All model rules take effect only in a small, local environment: the largest area a cell will search before taking an action is that encompassing its immediate neighbors. Similarly, local concentrations of mediators (rather than total amount in a simulation) are used to determine macrophage state changes, tissue damage, and all other functions that are mediator-driven. Tissue damage itself is a local concept: each cell has a health score that is factored in to its individual behavior. However, when collections of cells arranged in a tissue experience similar stimuli (e.g. pressure, local mediator concentrations, etc.) they will respond similarly and spatial patterns—like ulcers—can emerge. In addition, our model was calibrated solely to healthy tissue. Thus, it is notable that when pressure was applied, ulcers not only developed but did so in a comparable time frame to—and with morphology that mimicked—clinical outcomes: irregular shapes emerged despite being simulated as starting from a perfectly round ischemic area.

The PUABM was designed to synthesize phenomenological information into a framework that would allow hypothesis testing and might lead to deeper investigation of mechanisms of interest. We chose our parameter values and ranges to yield behaviors that were deemed relevant by both clinician and biologists on our interdisciplinary team. Many of the parameters that define key mechanisms of the model represent the cumulative actions of a set of cells or molecules, some of whose functions remain ill-defined. For this reason, almost all of the parameters values for the model were estimated based on the phenomena we wished to encode, rather than taking values estimated in the literature (which could not, by definition, exist). For example, we calibrated epithelial cell parameters so that unperturbed tissue remained healthy for periods of time much longer than a simulation, as described in *Materials and Methods*. For inflammatory mechanisms, we chose thresholds for cell state changes such that at the population level several states could co-exist when appropriate and ulcer progression occurred on a relevant time scale. Sampling distributions were similarly unknown, and so we reasoned that we could understand the broadest range of model capabilities by sampling parameters from uniform or log-uniform distributions. Our 1NN and re-simulations yielded different estimates of the time point of bifurcation. The re-simulations had much more input information, and there is likely something in the model that was not included as a feature in 1NN that could have improved its classification time. Mediators were used in 1NN because they could conceivably be measured at the bedside, but identification of other, potentially diagnostic, features would certainly improve our understanding of this phenomenon. In addition, to address hypotheses of inside-out ulcer formation, it will be important to develop a three-dimensional version of this model that will be able to simulate wounds with depth penetrating multiple layers of tissue.

The PUABM also represents an extension of our prior work, in which we created a hybrid equation- and agent-based model that simulates blood flow along with skin injury, inflammation, and ulcer formation [25]. As in the PUABM, the relationship between pressure and the course of ulcer formation was demonstrated in that prior study. The equation-based portion of that hybrid model was calibrated to data related to blood flow following experimental pressure responses in non-injured human subjects or to data from people with SCI, predicting a higher propensity to form PU in response to pressure in people with SCI vs. non-injured control subjects (both as cohorts and individual patients [25]). Thus, blood flow data could be integrated with clinical images to further improve diagnosis or treatment, in essence comprising a novel, multi-scale diagnostic platform for post-SCI ulcer formation.

In conclusion, we suggest that the primary value of the PUABM lies in its ability to recapitulate a broad range of pathology with qualitative, and at times quantitative, fidelity based on a single set of parameters, as well as broadly reproducing the full range of outcomes upon variation of only a few parameters. Better measurements at the bedside and further analysis may eventually allow the model be used to identify individual wound healing phenotypes and trajectories, determine appropriate treatment course, and design and test new treatment regimens.

## Supporting Information

S1 Text Model Pseudocode. (DOCX)

S1 Code SPARK code for PUABM. (ZIP)

S1 Table Parameters, descriptions, and default values. (DOCX)

S2 Table Information criteria outcomes for GMM Fit. (DOCX)

S1 Fig Bar graph of Tissue Damage at 10, 15, and 20 days post-SCI. For simulations in the upper plot, Pressure Cycle length was 2, 6, or 12h of ischemia followed by and equal period of reperfusion. In most simulations, ulceration occurs between day 15 (360 h) and day 20 (480 h). After ulceration, simulations with shorter pressure cycle lengths have more tissue damage initially. For comparison, the lower plot shows in vivo results [37] demonstrating that increasing the amount of ischemia increases damage (1h v 2h, 5 cycles) but for a given amount of ischemia, increasing the number of reperfusion events also increases damage (2h, 5 cycles v 1h, 10 cycles).
(DOCX)

S2 Fig Histogram of ulceration versus % initial injury. A 30% initial injury leads to ulceration roughly 50% of the time.
(DOCX)

S3 Fig Histogram and GMM Estimate of tissue damage at t = 1000 for 30% initial injury. Two disparate levels of tissue damage suggest two outcomes from simulations starting with the same initial conditions. Lower tissue damage is associated with no ulcer formation.
(DOCX)

S4 Fig Time courses for each feature in resolved versus ulcerated simulations. Blue time courses are from simulations that led to ulcers whereas red did not. Total counts of cells, mediators, and tissue health are plotted for 1000 ticks. Time courses of some features are very similar for both outcomes, but for other the differences are stark.
(DOCX)

S5 Fig 1NN over individual features. Classification accuracy (resolved versus ulcerated) for 1NN over each feature individually. Features were input as time vectors, using either all previous time points or the previous 50 ticks.
(DOCX)

S6 Fig Implementation of corticosteroid treatment in model rules. Local concentrations of corticosteroid functionally disable macrophages and neutrophils in the model, causing them to no longer produce or respond to mediators.
(DOCX)

S7 Fig Implementation of anti-DAMPs antibody in model rules. Anti-DAMPs antibodies are simulated as removing DAMPs from the model, lowering local concentrations in a quenching reaction.
(DOCX)
