# Supplementary material for: A Computational, Tissue-Realistic Model of Pressure Ulcer Formation in Individuals with Spinal Cord Injury
Source: PLoS Comput Biol. 2015 Jun 25;11(6):e1004309. doi: 10.1371/journal.pcbi.1004309 (PMC4482429; doi:10.1371/journal.pcbi.1004309)
Supplement: S1 Text — (DOCX) [file pcbi.1004309.s001.docx]

S1 Text. PUABM Pseudocode

Key:

**AGENTS**

**properties of agents**

*Data Layers –* In rules, this refers to the local value of a data layer

*User-defined-parameters*

functions-defined-inside-PUABM

SPARK-UNIVERSAL-FUNCTIONS

∝: “is proportional to”

At tick t, do the following:

- Reset tissue-health = 0
- Reset total-damage = 0
- If t MOD *shear-interval* == 0, apply or release shear
- Data Layers diffuse: *TNF*, *TGF*, *IL-1B*, *O_2_*, *DAMPs*, *ROS, Steroid, AntiDamps*
- Data Layers evaporate: *TNF*, *TGF*, *IL-1B*, *O_2_,* *DAMPs*, *Oxidase*, *ROS, Antioxidant, Steroid, AntiDamps*
- If t MOD *pressure-interval* == 0, apply or release pressure by changing values of data layer, *Pressure*
- In a randomly determined order,
  - **Epithelial Cells** make-step:
    - **border** = false
    - **velocity** = 0
    - If t > *antidamps-onset* and [(t – *antidamps-onset*) MOD *antidamps-reapply* == 0], set *AntiDamps* = *antidamps-dose*
    - If **alive**, epithelial-function:
      - Set *Antioxidant* = *antiox-level*
      - If *O_2_* < 0.75, increase *Oxidase* 0.01
      - If *O_2_* > 0 and *Oxidase* > 0, make *ROS* ∝ *O_2_* × *Oxidase*
      - If *Antioxidant* > 0 and *ROS* > 0
        - decrease *ROS* ∝ *Antioxidant* × *ROS*
        - decrease *Antioxidant* ∝ *Antioxidant* × *ROS*
      - If *AntiDamps* > 0 and *DAMPs* > 0: with probability *damps-bind*
        - Decrease *DAMPs* ∝ *AntiDamps* × *DAMPS*
        - Decrease *AntiDamps* ∝ *AntiDamps* × *DAMPs*
      - If *ROS* > 0, increase *injury-index* ∝ *ROS*
      - If *injury-index* > *injury-threshold* decrease **life** 90%
      - Decrease **life** ∝ (shear force)^2^
      - Decrease **life** ∝ *TNF*, *IL-1B*, 1 - *O_2_*
      - Increase **life** ∝ *TGF*, *O_2_*
      - Set **color**
      - If **life** < 0
        - Increase total-damage 1
        - **alive** = false
        - DIE, EXIT
      - **life** > 100 🡪 100
      - If **life** < 100, increase total-damage ∝ **life**
      - Increase tissue-health ∝ **life**
      - Increase *DAMPS* ∝ exp(-**life**/100)
    - Else,
      - If neighbors **alive**, **border** = true
      - Increase total-damage 1
  - **Blood Vessels** make-step:
    - **size** scales ∝ (1 - *pressure*)^4^
    - If one or fewer neighboring **Epithelial Cells** are **alive**, die
    - HATCH one **Macrophage** with probability ∝ **size**
    - HATCH one **Neutrophil** with probability ∝ **size**
    - If d > 0.0001, HATCH one **Neutrophil**
    - Increase *OXYGEN* ∝ **size**
    - If t > *steroid-onset*, set *Steroid* = *steroid-dose*
  - **Macrophages** make-step:
    - If *Steroid* > *mac-steroid-toxicity*
      - if **state** ==1, decrease activated-macrophages1 by 1
      - if **state** ==2, decrease activated-macrophages2 by 1
      - DIE, EXIT
    - Else:
      - WIGGLE 45°
      - JUMP
      - SNIFF *TGF* with probability 0.5
    - Else, with probability 0.3:
      - SNIFF *TNF*, then
      - SNIFF *IL-1B*
    - Else don’t SNIFF anything
    - If *TNF* > *tnf-activation-mac* or *IL-1B* > *il1-activation-mac*
      - **state** = 1 with probability = 1/(1 + *il1-activation-mac*)
      - increase activated-macrophages1
      - **color** = red
      - if previous **state** ==2, decrease activated-macrophages2 by 1
    - If *TGF* > *tgf-activation-mac* or *IL-1B* > il1-m2-activation
      - **state** = 2 with probability = 1/(1 + *tgf-activation-mac*)
      - increase activated-macrophages2
      - **color** = green
      - if previous **state** ==1, decrease activated-macrophages1 by 1
    - decrease **age** 1
    - if **age** < 0
      - if **state** ==1, decrease activated-macrophages1 by 1
      - if **state** ==2, decrease activated-macrophages2 by 1
      - DIE, EXIT
  - **Neutrophils** make-step:
    - If *Steroid* > *pmn-steroid-toxicity*
      - increase *ROS* 0.001
      - if **state** ==1, decrease activated-neutrophils 1
      - DIE, EXIT
    - Else:
      - WIGGLE 45°
      - JUMP
      - SNIFF *DAMPs*
      - If **state** == 0 and *DAMPS* > *danger-pmn-activation*
        - **state** = 1
        - **color** = black
        - Increase **age** 3-8 ticks
      - If **state** == 1, increase *TNF* ∝ (1+*TNF*)/(1+*TGF*)
      - If **state** == 0
        - If *TGF* < 0.001

survey **Epithelial Cells** in a neighborhood of radius 1

if their mean **life** < 90, **state** == 1

- - - - decrease **age** 1
      - if **age** < 0
        - increase *ROS* 0.001
        - if **state** == 1, decrease activated-neutrophils
        - DIE, EXIT
      - Query values of: total-TNF, total-TGF, total-danger, total-pressure, total-oxygen, blood-flow, total-antioxidant, total-antidamps, total-ROS, total-oxidase, activated-macrophages1, activated-macrophages2, activated-neutrophils, epithelial-number
