# Supplementary material for: A Computational, Tissue-Realistic Model of Pressure Ulcer Formation in Individuals with Spinal Cord Injury
Source: PLoS Comput Biol. 2015 Jun 25;11(6):e1004309. doi: 10.1371/journal.pcbi.1004309 (PMC4482429; doi:10.1371/journal.pcbi.1004309)
Supplement: S1 Table — (DOCX) [file pcbi.1004309.s003.docx]

**S1 Table**

| **parameter** | **description** | **default value or range** |
| --- | --- | --- |
| initial-pressure | pressure intensity, varies in [0,1] | 0.5 |
| pressure-interval | half of period of one pressure cycle | 2 |
| macrophage-production | rate of resting macrophages arriving via open blood vessels | 0.5 |
| neutrophil-production | rate of resting neutrophils arriving via open blood vessels | 0.5 |
| oxygen-production | number of oxygen packages released by each blood vessel on each tick | 2 |
| pmn damps recruitment | danger signal threshold above which neutrophils are recuited | 0.0001 |
| epi nbr til dead | number of adjacent epithelial cells below which bv no longer exists | 1 |
| hyperemia | threshold of oxygen value on previous tick to induce hyperemia when pressure is released | 0.5 |
| max-size | Radius of blood vessel when open (pressure not applied) | 0.6-0.8 |
| blood vessel constrict exponent | exponent of radial decrease in bv proportional to pressure applied | 4 |
| mac lifespan | random variable defining maximal number of ticks a mac will live | 100-150 |
| macrophage-speed | rate factor for jump and sniff functions | 0.2 |
| mac jump | distance mac will jump in one tick during random movement | 0.333 |
| mac sniff | rate of chemoattraction, scaled by mac-speed | 0.1 |
| il1-mac-activation | local concentration of il1-B above which M2 and resting macs shift to M1 | 0.2 |
| il1-m2-activation | local concentration of il1-B above which M1 and resting macs shift to M2 | 0.6 |
| tnf-mac-activation | local concentration of tnf-a above which M2 and resting macs shift to M1 | 0.15 |
| tgf-mac-activation | local concentration of tgf-B above which M1 and resting macs shift to M2 | 0.2 |
| tgf-production-mac | scalar coefficient of amount of tgf-B released by M2 macrophages on each tick | 0.04 |
| il1-production-mac | scalar coefficient of amount of il1-B released by M1 macrophages on each tick | 0.02 |
| pmn lifespan | random variable defining maximal number of ticks a neutrohil will live | 10 to 20 |
| pmn bonusLife | random variable defining ticks added to neutrophil lifespan upon activation | 5 to 15 |
| pmn jump | distance neutrophil will jump in one tick during random movement | 3 |
| pmn-attract | scale macrophage-speed to be applicable to neutrophils | 0.15 |
| pmn wiggle | maximal angle of direction change in one tick during random movement | 45 |
| tgf-pmn-suppression | threshold of tgf under which neutrophils may be activated if other conditions are met | 0.001 |
| local life activation | if average of neighbors health is below this threshold, neutrophils activate | 90 |
| danger-pmn-activation | local concentration ofdanger signal above which neutrophils are activated | 0.01 |
| radical leakage on death | amount of ROS secreted by dying neutrophils | 0.001 |
| initial life | life score of epithelial cells at the beginning of the simulation | 100 |
| healing-rate | upper bound for rv heal, scales effects of TGF on life score | 0.02 |
| tgf-epithelial | scale tgf effect on life score | 0.5 |
| tnf-epithelial | scale tnf effect on life score | 0.8 |
| damps production rate by epithelial cells | scale epithelial life score by this factor; add damps according to exp -life*prod_rate | 0.1 |
| minimum healthy oxygen | threshold of oxygen under which oxidase is produced | 0.75 |
| oxidase incrementor | amount of oxidase produced each tick if o2 is under minimum healthy oxygen | 0.01 |
| radical-injury | amount of local ROS that counts toward ROS injury score | 0.4 |
| injury-threshhold | after ROS injury passes this level, cell heath decreases by ros life decrement | 0.8 |
| ros life decrement | amount of life remaining after ros passes injury-threshold | 0.1 |
| healthy tissue rgb | rbg triplet defining color of health epithelial cellls in the visualization | [0.93,0.83,0.71] |
| damaged tissue rgb | rgb triplet defining color of unhealthy epithelial cells | [1,0,0] |
| o2-epithelial | sensitivity of epithelial cell health to oxygen levels | 0.2 |
| oxidase evaporate | linear degradation coefficient of data layer on each tick | 0.75 |
| tnf evaporate | linear degradation coefficient of data layer on each tick | 0.85 |
| tgf evaporate | linear degradation coefficient of data layer on each tick | 0.85 |
| il-1b evaporate | linear degradation coefficient of data layer on each tick | 0.85 |
| o2 evaporate | linear degradation coefficient of data layer on each tick | 0.95 |
| ros evaporate | linear degradation coefficient of data layer on each tick | 0.85 |
| danger signal evaporate | linear degradation coefficient of data layer on each tick | 0.95 |
| antiox evaporate | linear degradation coefficient of data layer on each tick | 0.85 |
| steroid evaporate | linear degradation coefficient of data layer on each tick | 0.85 |
| damps-clear | linear degradation coefficient of data layer on each tick | 0.85 |
| oxygen-diff | square root of diffusion coefficient for oxygen each tick | 0.9 |
| ros diffuse | square root of diffusion coefficient each tick | 0.3 |
| il-1b diffuse | square root of diffusion coefficient each tick | 0.9 |
| tgf diffuse | square root of diffusion coefficient each tick | 0.9 |
| tnf diffuse | square root of diffusion coefficient each tick | 0.9 |
| danger signal diffuse | square root of diffusion coefficient each tick | 0.9 |
| steroid diffuse | square root of diffusion coefficient each tick | 0.9 |
| antidamps diffuse | square root of diffusion coefficient each tick | 0.9 |
| mac-steroid-toxicity | local concentration of steroid above which macrophages die | 0.8 |
| pmn-steroid-toxicity | local concentration of steroid above which neutrophils die | 0.8 |
| antidamps-onset | tick at which anti-damps antibody is first applied | 0 |
| antidamps-dose | level at which anti-damps antibody is applied | 0 |
| antidamps-reapply | number of ticks between re-applications of anti-damps antibodies | 250 |
