# Supplementary material for: A Computational, Tissue-Realistic Model of Pressure Ulcer Formation in Individuals with Spinal Cord Injury
Source: PLoS Comput Biol. 2015 Jun 25;11(6):e1004309. doi: 10.1371/journal.pcbi.1004309 (PMC4482429; doi:10.1371/journal.pcbi.1004309)
Supplement: S2 Table — (DOCX) [file pcbi.1004309.s004.docx]

S2 Table. Information Criteria for fitting a mixture of 1-4 Gaussians to the Total Damage data for Default Parameters (including pressure).

|  | **Number of Independent Gaussians** | | | |
| --- | --- | --- | --- | --- |
|  | **1** | **2** | **3** | **4** |
| **AIC** | 11959.9 | 11723.8 | 11728.9 | 11735.1 |
| **BIC** | 11969.7 | 11748.4 | 11768.2 | 11789.1 |
