# Supplementary figures and images for: A Computational, Tissue-Realistic Model of Pressure Ulcer Formation in Individuals with Spinal Cord Injury
Source: PLoS Comput Biol. 2015 Jun 25;11(6):e1004309. doi: 10.1371/journal.pcbi.1004309 (PMC4482429; doi:10.1371/journal.pcbi.1004309)

**S1 Figure**


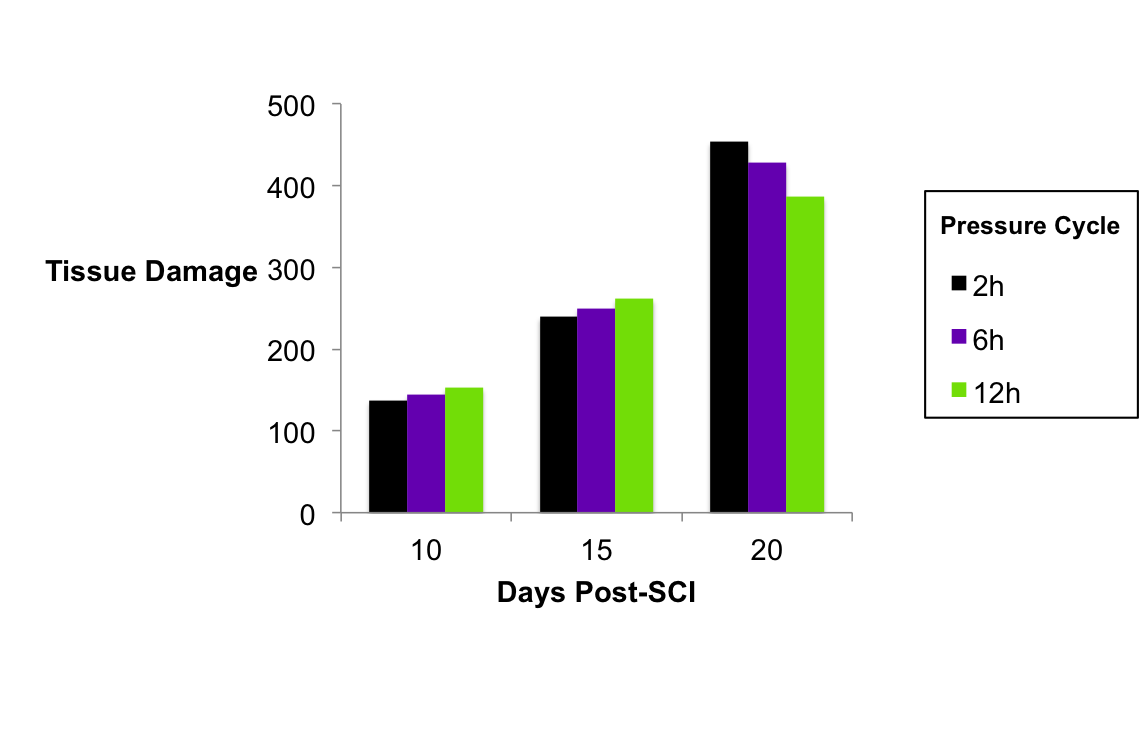


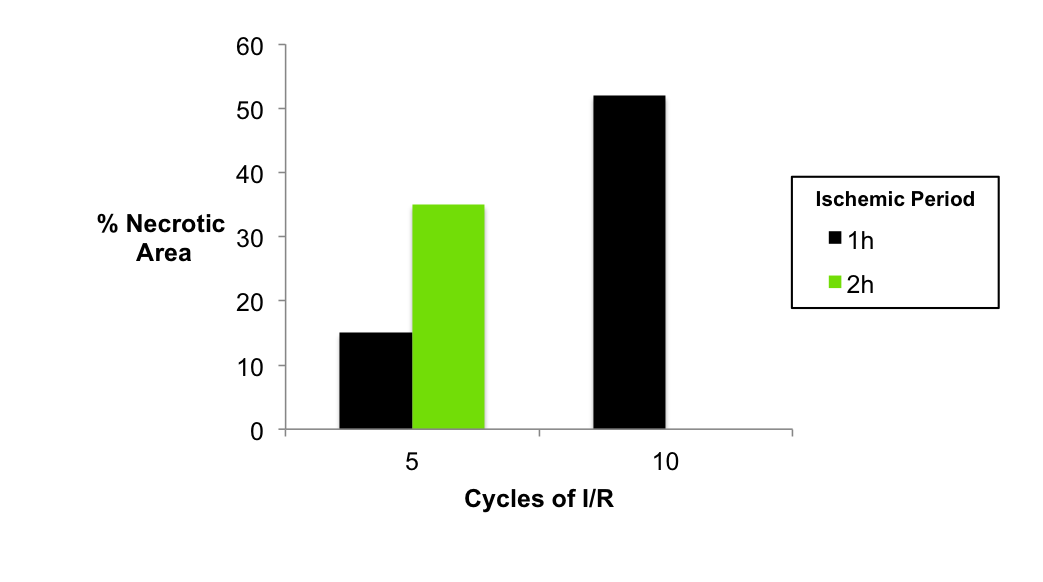

Supplement: S1 Fig — For simulations in the upper plot, Pressure Cycle length was 2, 6, or 12h of ischemia followed by and equal period of reperfusion. In most simulations, ulceration occurs between day 15 (360 h) and day 20 (480 h). After ulceration, simulations with shorter pressure cycle lengths have more tissue damage initially. For comparison, the lower plot shows in vivo results [37] demonstrating that increasing the amount of ischemia increases damage (1h v 2h, 5 cycles) but for a given amount of ischemia, increasing the number of reperfusion events also increases damage (2h, 5 cycles v 1h, 10 cycles). (DOCX) [file pcbi.1004309.s005.docx]

**S2 Figure**


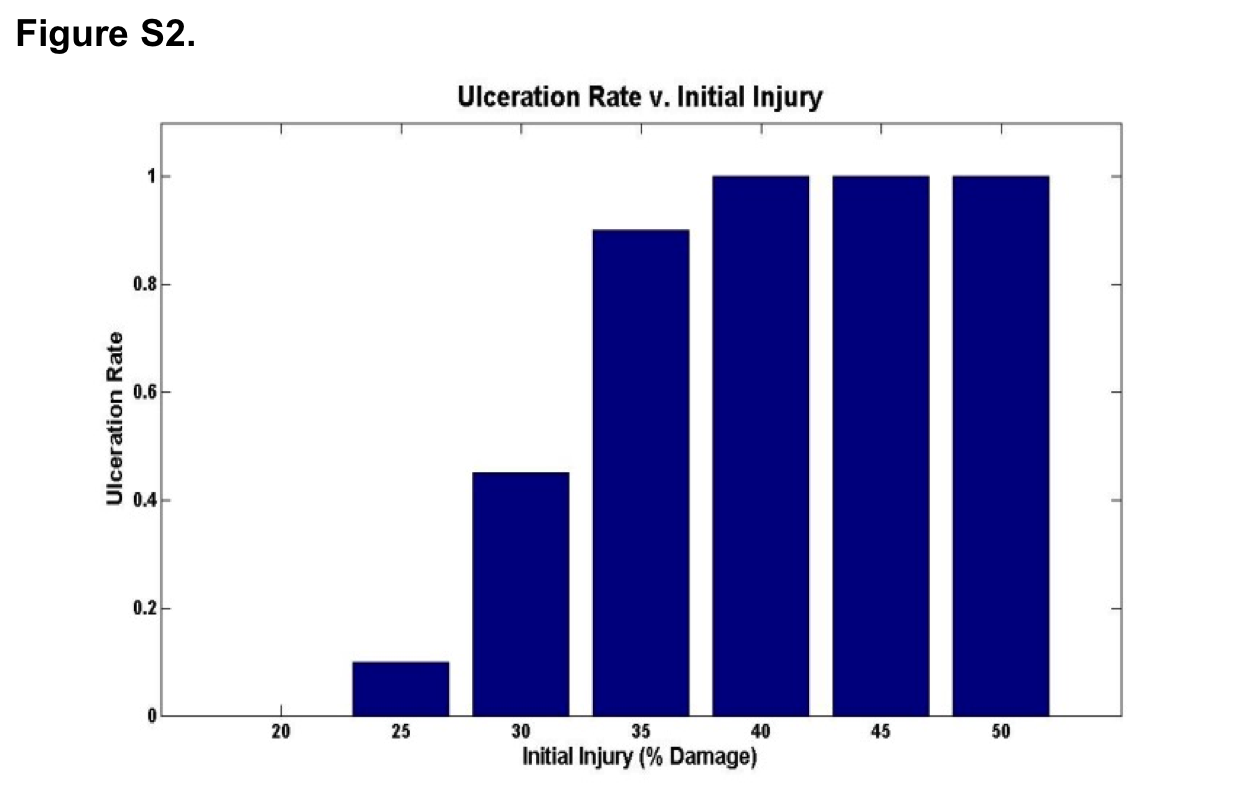

Supplement: S2 Fig — A 30% initial injury leads to ulceration roughly 50% of the time. (DOCX) [file pcbi.1004309.s006.docx]

**S3 Figure**


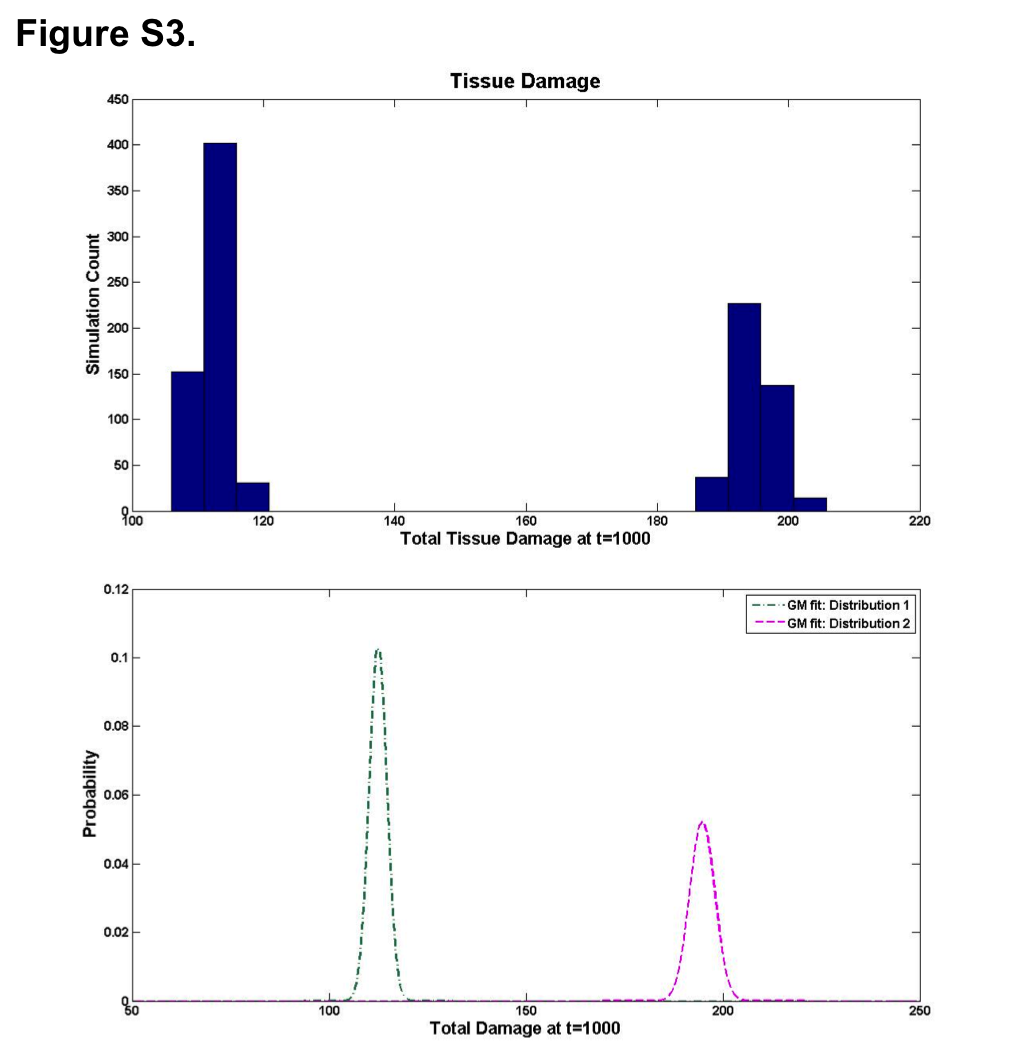

Supplement: S3 Fig — Two disparate levels of tissue damage suggest two outcomes from simulations starting with the same initial conditions. Lower tissue damage is associated with no ulcer formation. (DOCX) [file pcbi.1004309.s007.docx]

**S4 Figure**


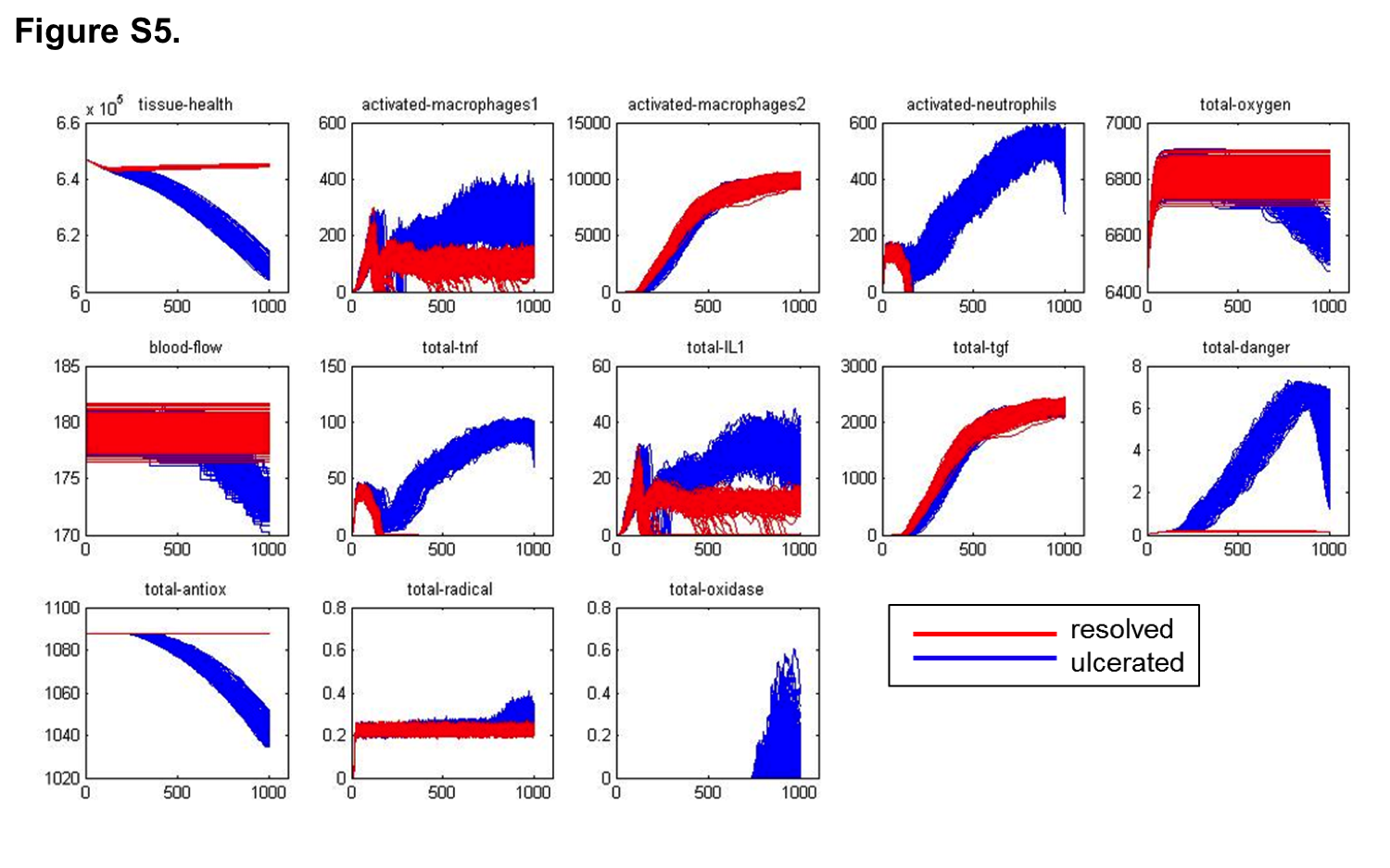

Supplement: S4 Fig — Blue time courses are from simulations that led to ulcers whereas red did not. Total counts of cells, mediators, and tissue health are plotted for 1000 ticks. Time courses of some features are very similar for both outcomes, but for other the differences are stark. (DOCX) [file pcbi.1004309.s008.docx]

**S5 Figure**


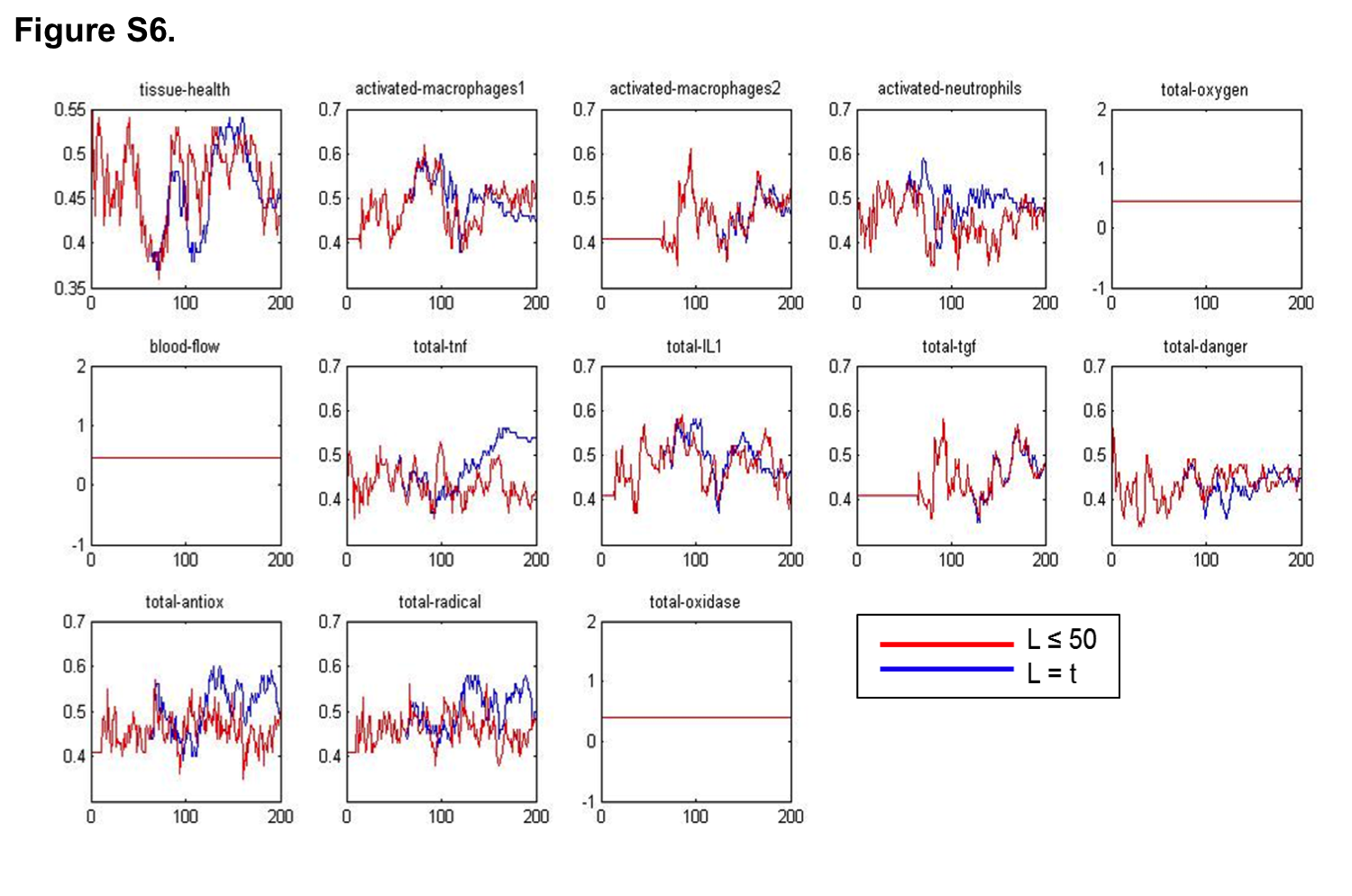

Supplement: S5 Fig — Classification accuracy (resolved versus ulcerated) for 1NN over each feature individually. Features were input as time vectors, using either all previous time points or the previous 50 ticks. (DOCX) [file pcbi.1004309.s009.docx]

**S6 Figure**


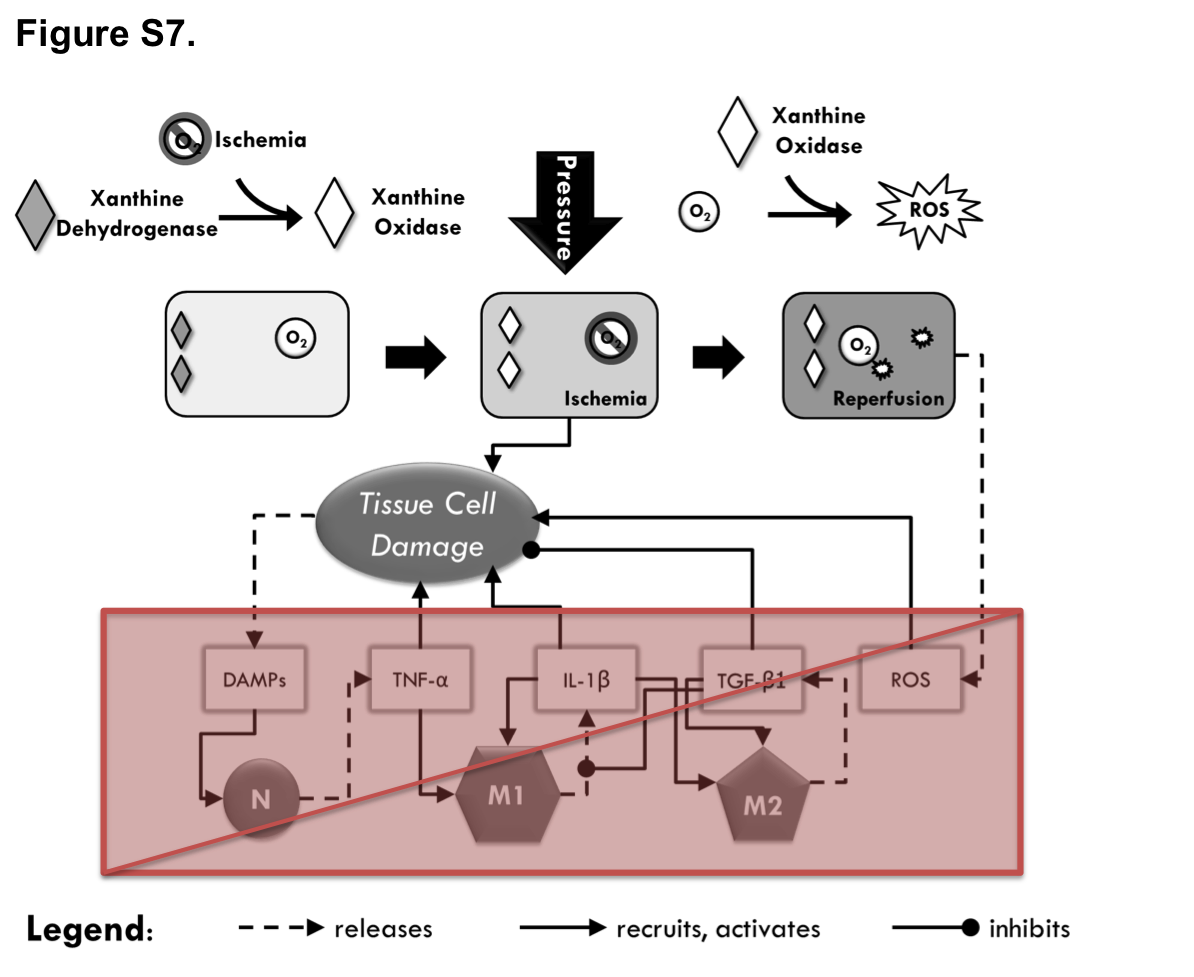

Supplement: S6 Fig — Local concentrations of corticosteroid functionally disable macrophages and neutrophils in the model, causing them to no longer produce or respond to mediators. (DOCX) [file pcbi.1004309.s010.docx]

**S7 Figure**


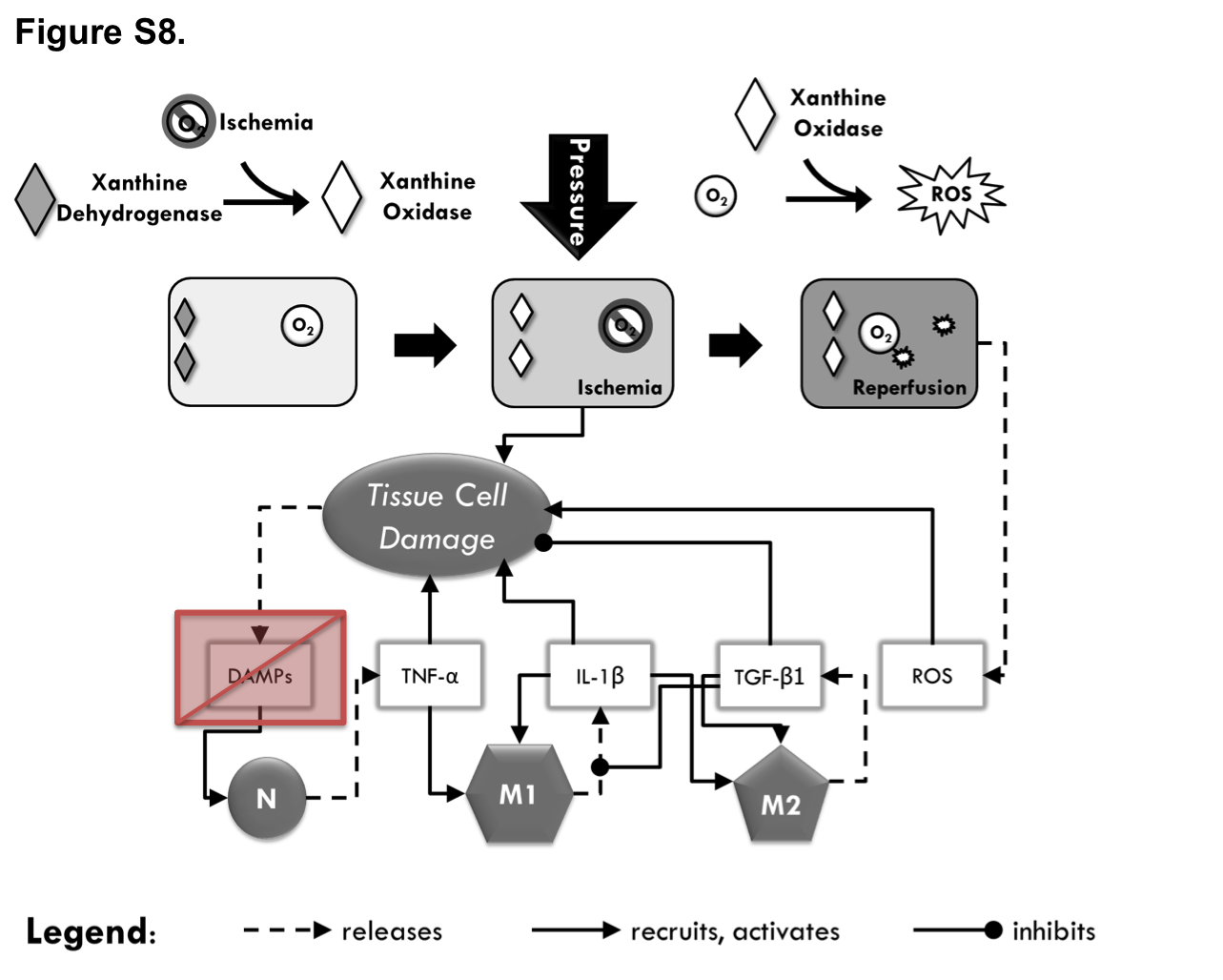

Supplement: S7 Fig — Anti-DAMPs antibodies are simulated as removing DAMPs from the model, lowering local concentrations in a quenching reaction. (DOCX) [file pcbi.1004309.s011.docx]
